# *In situ* small-angle X-ray scattering studies during the formation of polymer/silica nanocomposite particles in aqueous solution†

**DOI:** 10.1039/d1sc03353k

**Published:** 2021-10-20

**Authors:** A. Czajka, G. Liao, O. O. Mykhaylyk, S. P. Armes

**Affiliations:** Department of Chemistry, University of Sheffield Dainton Building, Brook Hill Sheffield South Yorkshire S3 7HF UK adam.czajka@mail.co.uk s.p.armes@sheffield.ac.uk

## Abstract

This study is focused on the formation of polymer/silica nanocomposite particles prepared by the surfactant-free aqueous emulsion polymerization of 2,2,2-trifluoroethyl methacrylate (TFEMA) in the presence of 19 nm glycerol-functionalized aqueous silica nanoparticles using a cationic azo initiator at 60 °C. The TFEMA polymerization kinetics are monitored using ^1^H NMR spectroscopy, while postmortem TEM analysis confirms that the final nanocomposite particles possess a well-defined core–shell morphology. Time-resolved small-angle X-ray scattering (SAXS) is used in conjunction with a stirrable reaction cell to monitor the evolution of the nanocomposite particle diameter, mean silica shell thickness, mean number of silica nanoparticles within the shell, silica aggregation efficiency and packing density during the TFEMA polymerization. Nucleation occurs after 10–15 min and the nascent particles quickly become swollen with TFEMA monomer, which leads to a relatively fast rate of polymerization. Additional surface area is created as these initial particles grow and anionic silica nanoparticles adsorb at the particle surface to maintain a relatively high surface coverage and hence ensure colloidal stability. At high TFEMA conversion, a contiguous silica shell is formed and essentially no further adsorption of silica nanoparticles occurs. A population balance model is introduced into the SAXS model to account for the gradual incorporation of the silica nanoparticles within the nanocomposite particles. The final PTFEMA/silica nanocomposite particles are obtained at 96% TFEMA conversion after 140 min, have a volume-average diameter of 216 ± 9 nm and contain approximately 274 silica nanoparticles within their outer shells; a silica aggregation efficiency of 75% can be achieved for such formulations.

## Abstract 2

SAXS is used to study the formation of polymer/silica nanocomposite particles prepared by surfactant-free aqueous emulsion polymerization of 2,2,2-trifluoroethyl methacrylate in the presence of silica nanoparticles using a azo initiator at 60 °C.

## Graphical abstract

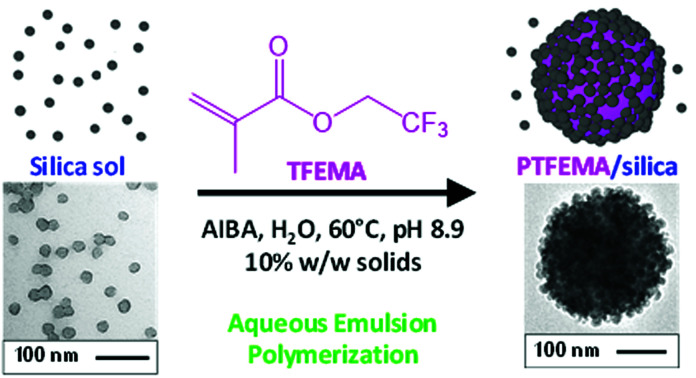

## Introduction

Ultrafine aqueous silica sols have been manufactured on an industrial scale by various chemical companies for many decades.^1^ They have been used for a wide range of applications, including scratch-resistant and anti-reflective coatings,^2,3^ corrosion protection,^4^ and Pickering emulsifiers.^5^ It is well-established that conducting polymerizations in the presence of such silica sols enables the preparation of polymer/silica nanocomposite particles under suitable conditions.^6–11^ In such syntheses, the insoluble polymer chains adsorb at the surface of the silica nanoparticles, leading to their controlled heteroflocculation.^6,12,13^ The surface of the final polymer/silica particles is silica-rich, which accounts for their colloidal stability.^14,15^ For example, in 1974 Iler and McQueston demonstrated that micrometer-sized nanocomposite particles could be obtained *via* copolymerization of either urea or melamine with formaldehyde in the presence of a 50 nm silica sol.^16^ Such microporous particles were evaluated as a stationary phase for liquid chromatography columns. In 1992 Gill and co-workers reported the synthesis of polyaniline/silica nanocomposite particles *via* oxidative polymerization of aniline in the presence of a commercial ultrafine silica sol.^17^ The same approach was subsequently extended to include polypyrrole/silica and poly(3,4-ethylenedioxythiophene)/silica nanocomposite particles.^11,18^ Such highly coloured particles have been evaluated for the development of immunodiagnostic assays.^19^ Moreover, their electrical conductivity means that they can efficiently accumulate surface charge and hence be accelerated up to hypervelocities using a high-field van der Graaf accelerator. Hence they have been reported to be useful synthetic mimics for silica-rich cosmic dust in space science experiments.^20,21^

Over the past two decades or so, the polymerization of various vinyl monomers in the presence of silica sols has led to a new class of colloidal nanocomposite particles that offer a range of interesting applications.^10,22,23^ For example, Fujii *et al.* reported that poly(4-vinylpyridine)/silica nanocomposite particles can act as pH-responsive Pickering emulsifiers for oil-in-water emulsions.^5,24,25^ Amalvy and co-workers showed that film-forming colloidal nanocomposite particles could be obtained by copolymerizing *n*-butyl acrylate with 4-vinylpyridine.^26^ In this case, the latter comonomer ensures a strong acid–base interaction between the copolymer chains and the silica nanoparticles, which is essential for nanocomposite formation.^27^ Inspired by such prototype formulations, a team of BASF scientists developed film-forming polymer/silica nanocomposite particles as the key component in a dirt-shedding architectural exterior paint formulation that is sold throughout continental Europe.^28^ Similarly, scientists at The Cabot Corporation designed highly crosslinked copolymer/silica nanocomposite particles that act as mechanically durable ‘spacer’ particles for laser toners.^29^ There are at least two examples of successful commercial exploitation based on colloidal nanocomposite particles prepared by *in situ* copolymerization of vinyl monomers in the presence of an ultrafine silica sol.

It is also possible to produce vinyl polymer/silica nanocomposite particles by surface modification of the silica sol, rather than by using an auxiliary comonomer such as 4-vinylpyridine. For example, commercially available glycerol-functionalized ultrafine silica sols^30^ were used by Schmid *et al.* to prepare either polystyrene/silica or poly(styrene-co-*n*-butyl acrylate)/silica nanocomposite particles.^9,12^ In the latter case, the well-defined core–shell morphology of the original nanocomposite particles leads to the formation of a 3D honeycomb structure within dried films comprising interconnected silica nanoparticles embedded within a copolymer matrix. This approach was later extended to include an all-acrylic film-forming composition by Fielding and co-workers, which resulted in highly transparent nanocomposite coatings.^31^

**Scheme 1:**
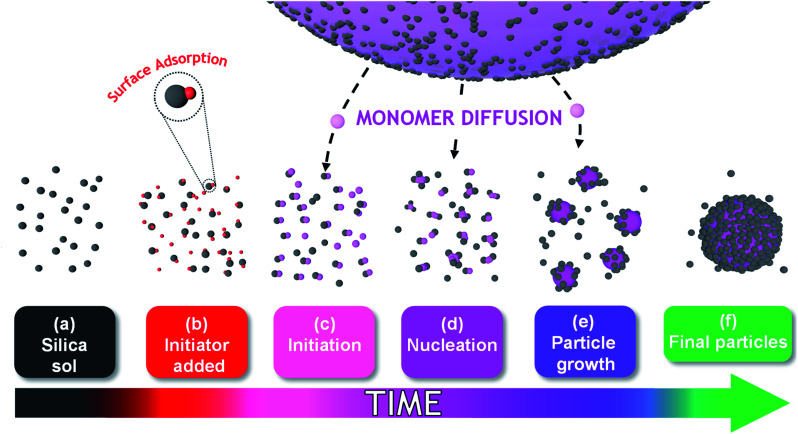
The proposed mechanism of formation of nanocomposite particles during aqueous emulsion polymerization of a water-immiscible monomer (TFEMA) in the presence of silica nanoparticles. (a) Initial glycerol-functionalized anionic silica nanoparticles. (b) Addition of AIBA (denoted as red spheres) leads to electrostatic adsorption of some of this cationic initiator onto the anionic silica nanoparticles, with the rest remaining in the aqueous continuous phase. (c) Surface polymerization of TFEMA produces hydrophobic patches of PTFEMA on the silica nanoparticles. (d) Incipient flocculation of the PTFEMA-coated silica nanoparticles produces PTFEMA/silica aggregates. (e) TFEMA diffuses from the giant monomer-droplets into these nascent nuclei, which become monomer-swollen and grow in size. (f) PTFEMA/silica nanocomposite particles are produced with a well-defined core–shell morphology.

The mechanism of particle formation during such colloidal nanocomposite syntheses has been investigated by Schmid *et al.*,^12^ Fielding *et al.*,^31^ and also by Bon and co-workers, see Scheme 1 for a schematic representation.^32–34^ However, such studies typically involve periodic sampling of the reaction mixture, followed by quenching of the polymerization and postmortem analysis at ambient temperature using analytical techniques such as transmission electron microscopy (TEM), dynamic light scattering (DLS) or disk centrifuge photosedimentometry (DCP). As far as we are aware, no *in situ* scattering studies of the development of the nanocomposite particle morphology have been conducted during the polymerization. One obvious reason for this omission is the difficulty of performing such experiments on inherently heterogeneous reaction mixtures, particularly when water-immiscible vinyl monomers are involved.

**Fig. 1:**
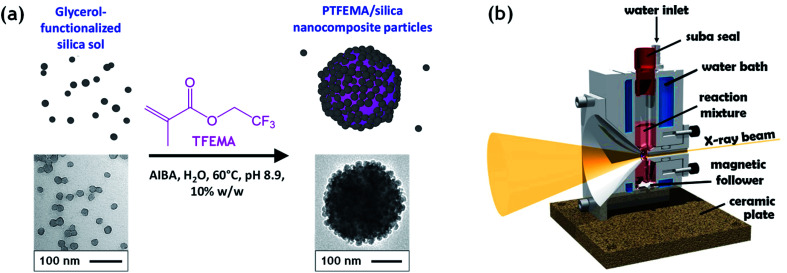
(a) Schematic representation (plus TEM images) of the synthesis of PTFEMA/silica nanocomposite particles by the surfactant-free aqueous emulsion polymerization of 2,2,2-trifluoroethyl methacrylate (TFEMA) using a cationic azo initiator (AIBA) at 60 °C in the presence of a commercial 19 nm glycerol-functionalized aqueous silica sol (Bindzil CC401). The latter anionic nanoparticles form a particulate shell at the surface of the PTFEMA latex cores and hence confer colloidal stabilization. (b) Schematic cross-section of the bespoke stirrable reaction cell used. The volume of the reaction solution within this cell is approximately 2.0 mL, which is sufficient to enable postmortem analysis of the final nanocomposite particles after performing time-resolved small-angle X-ray scattering (SAXS) experiments.

Herein, we utilize a recently reported stirrable reaction cell^35,36^ to conduct the first *in situ* small-angle X-ray scattering studies during the formation of vinyl polymer/silica colloidal nanocomposite particles, see Fig. 1. More specifically, such colloidal nanocomposite syntheses involve the surfactant-free aqueous emulsion polymerization of 2,2,2-trifluoroethyl methacrylate (TFEMA) in the presence of an ultrafine glycerol-functionalized aqueous silica sol. This semi-fluorinated vinyl monomer was selected because it offers much stronger X-ray contrast than conventional vinyl monomers such as styrene.^37^ This enables high-quality SAXS patterns to be collected within short time frames, which is essential to provide new insights into the mechanism of particle nucleation and growth.

## Results and discussion

### Nanocomposite syntheses and kinetic data

Well-defined colloidally stable nanocomposite particles were prepared by the surfactant-free aqueous emulsion polymerization of TFEMA in the presence of a glycerol-functionalized silica sol (Bindzil CC 401), see Fig. 1. This commercially available silica sol was kindly provided by Nouryon and prepared using a proprietary protocol described in the patent literature.^30^ It is supplied as a 40% w/w aqueous dispersion with a mean particle diameter of 19 nm. We have shown that various types of vinyl polymer/silica nanocomposite particles can be prepared over a wide range of conditions using such glycerol-functionalized silica sols.^9,12,21,38^ This versatile approach eliminates the need for auxiliary comonomers, added surfactants, or alcoholic silica sols.^9,12^ For example, Schmid *et al.* reported that well-defined polystyrene/silica nanocomposites are formed in the presence of Bindzil CC401 silica nanoparticles with particularly high silica aggregation efficiency.^12^ In this case, electrostatic adsorption of an cationic azo initiator (AIBA) onto the anionic silica nanoparticles is a prerequisite for nanocomposite particle formation and the silica nanoparticles adsorb onto the polymer latex cores to form contiguous shells that confer colloidal stability.^12^ We chose to use the same cationic initiator, polymerization temperature and essentially the same solution pH in the present study.

Previously we have investigated *in situ* nanocomposite formation by aqueous emulsion polymerization of common vinyl monomers such as styrene^12^ or methyl methacrylate.^31^ Herein we extend our studies of colloidal nanocomposite particles to include 2,2,2-trifluoroethyl methacrylate (TFEMA). This semi-fluorinated monomer has an aqueous solubility of approximately 2.9 g dm^−3^ at 25 °C,^39^ which is approximately an order of magnitude higher than that of styrene (0.31 g dm^−3^ at 25 °C). Nevertheless, the aqueous solubility of TFEMA is sufficiently low to ensure a genuine aqueous emulsion polymerization formulation. Furthermore, given that the *T*_g_ of PTFEMA homopolymer is around 55 °C, the nanoparticles retain their original morphology during TEM analysis. More importantly, the corresponding homopolymer, PTFEMA, scatters X-rays much more strongly than polystyrene and hence provides much better contrast with respect to the aqueous continuous phase during *in situ* SAXS studies.^40^

**Fig. 2:**
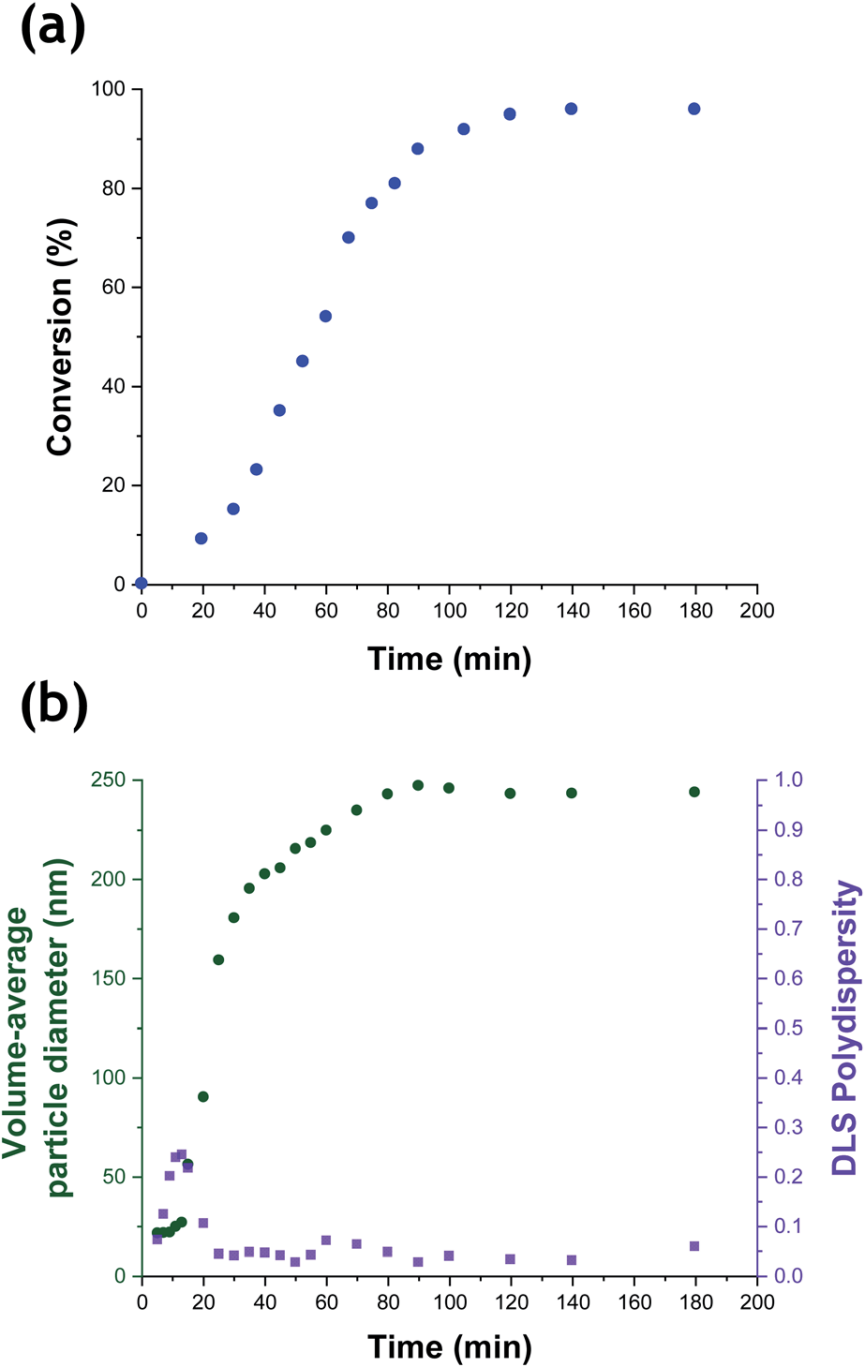
(a) Conversion *vs.* time curve obtained from ^1^H NMR spectroscopy studies (recorded for aliquots periodically extracted from the reaction mixture after quenching by dilution with CDCl_3_) for the laboratory-scale aqueous emulsion polymerization of TFEMA in the presence of a glycerol-functionalized silica sol (Bindzil CC401) using a cationic azo initiator at 60 °C targeting 10% w/w solids. (b) Evolution in volume-average particle diameter and polydispersity determined by dynamic light scattering studies of aliquots periodically extracted from the quenched reaction solution (diluted to 0.20% w/w prior to analysis using deionized water).

The kinetics of TFEMA polymerization and the concomitant evolution in particle size were monitored during a laboratory-scale synthesis (55 mL reaction volume) using the conditions shown in Fig. 1 by periodically withdrawing 1.0 mL aliquots from the reaction mixture for analysis. The polymerization was quenched by immediately immersing each aliquot in an ice bath with concomitant exposure of the reaction mixture to air. To monitor the evolution in particle size, 20 μL of each aliquot was diluted with deionized water (980 μL) to produce a series of 0.20% w/w aqueous dispersions for dynamic light scattering (DLS) analysis. Intensity-average size distributions can be converted into volume-average size distributions using Mie theory.^41^ This was performed using the software provided by the DLS instrument manufacturer. Instantaneous TFEMA conversions were determined by recording ^1^H NMR spectra for 50 μL aliquots extracted from the reaction solution (after dilution of each aliquot with 500 μL CDCl_3_ and using anhydrous MgSO_4_ to remove residual water). Fig. 2 shows the kinetics of TFEMA polymerization and the evolution in particle size observed during the laboratory-scale synthesis of PTFEMA/silica nanocomposites under the conditions shown in Fig. 1. During the early stages of the polymerization, DLS studies indicate a significant increase in particle diameter after 13 min, which likely corresponds to the initial stage of micellar nucleation. Furthermore, a well-defined maximum in DLS polydispersity is also observed at around 13 min. Thereafter, the DLS polydispersity remains relatively low (0.10). Between 13 and 35 min, there is a period of rapid particle growth. After the latter time point, the rate of particle growth decreases, but remains linear up to approximately 90 min. Interestingly, between 90 and 140 min there is a subtle reduction in the volume-average particle diameter. Given that there is a relatively large difference in density between TFEMA monomer (1.18 g cm^−3^) and PTFEMA (1.47 g cm^−3^),^42^ the dilatometric effect during TFEMA polymerization minimizes the increase in volume that occurs as the growing monomer-swollen particles are converted into PTFEMA/silica nanocomposite particles. This has been known to produce a reduction in particle size during the final stages of emulsion polymerization under ‘monomer-starved’ conditions, in which all of the remaining monomer is located within the monomer-swollen particles.^43^ This most likely accounts for the modest reduction in particle size observed towards the end of the TFEMA polymerization (*i.e.* between 90 and 140 min in Fig. 2b). Furthermore, the rate of monomer conversion decreases significantly after 80 min, see Fig. 2a. After 140 min, no further increase in either conversion or particle size was observed. At this time point, a volume-average particle diameter of 244 nm (DLS polydispersity = 0.03) was observed and ^1^H NMR studies indicated a final monomer conversion of 96%. TEM studies confirm the formation of well-defined core–shell PTFEMA/silica nanocomposite particles with an estimated number-average diameter of 240 nm, see Fig. S2a.†

According to Fig. 2, there is a significant increase in particle size during nucleation that occurs with relatively little change in monomer conversion. As noted by Schmid *et al.*,^12^ the cationic AIBA initiator is electrostatically adsorbed onto the anionic silica nanoparticles. Thus, thermal decomposition of this reagent leads to surface polymerization of the TFEMA, which produces hydrophobic PTFEMA patches on the nanoparticles. This leads to their incipient aggregation, which results in a significant increase in particle size with minimal change in monomer conversion. Subsequently, TFEMA monomer (aqueous solubility = 2.9 g dm^−3^ at 25 °C) diffuses from the monomer droplets into these ill-defined nascent nuclei and swells the adsorbed PTFEMA chains. The upturn in the rate of polymerization reflects the relatively high local TFEMA concentration within such monomer-swollen particles, which is also observed for conventional aqueous emulsion polymerization formulations.^44^

### Onset of micellar nucleation

**Fig. 3:**
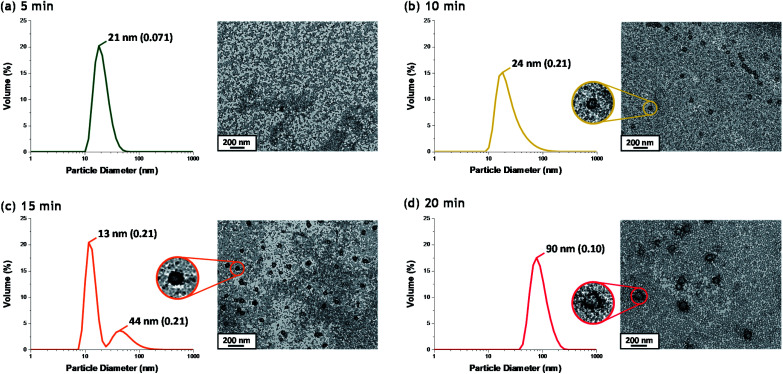
Volume-average size distributions and polydispersities determined by DLS and corresponding TEM images recorded during the first 20 min of the surfactant-free polymerization of TFEMA in the presence of 19 nm glycerol-functionalized silica nanoparticles using a cationic azo initiator at 60 °C after (a) 5 min; (b) 10 min; (c) 15 min; (d) 20 min.

Although there have been many reports of the synthesis and characterization of various vinyl polymer/silica colloidal nanocomposites,^10,22,26,45,46^ no studies have examined the nucleation event for such formulations. Given that such syntheses often utilize water-immiscible vinyl monomers, reliable sampling of the inherently heterogeneous reaction mixture presents intrinsic technical difficulties. Nevertheless, both prior studies^31^ and the DLS data shown in Fig. 2b confirm that such periodic sampling is feasible. Accordingly, Fig. 3 shows the calculated volume-average size distributions and corresponding TEM images obtained during the first 20 min of the TFEMA polymerization. After 5 min, only a unimodal size distribution that approximately corresponds to the 19 nm diameter of the original silica nanoparticles is discernible, see Fig. 3a. This volume-average diameter increases slightly after 10 min, with close inspection indicating a broader size distribution (as indicated by the significantly higher DLS polydispersity), see Fig. 3b. Indeed, the corresponding TEM image indicates the presence of a few nascent nuclei with estimated number-average diameters of approximately 40 nm. Subsequently, a bimodal DLS size distribution is observed after 15 min (Fig. 3c), with TEM analysis confirming the formation of significantly larger particles. Initially, there are only silica nanoparticles present and the DLS polydispersity is relatively low. Once nucleation occurs after 10–15 min, there are now two co-existing populations: the original 19 nm diameter silica nanoparticles plus an (increasing) proportion of ill-defined polymer/silica aggregates of approximately 24–44 nm diameter. This bimodal size distribution inevitably leads to a higher DLS polydispersity. Within 20 min, the polymer/silica aggregates become sufficiently large that they now dominate the light scattering (see Fig. 3c and d). This is because the scattered light intensity scales with the sixth power of the particle radius.^47^ Consequently, the particle size distribution effectively becomes unimodal so the DLS polydispersity falls (and remains relatively low) after this time point. Based on the above DLS and TEM observations, nucleation appears to occur within 10–15 min, which is consistent with the time frame of approximately 13 min corresponding to the upturn in the volume-average particle diameter and the local maximum in DLS polydispersity shown in Fig. 2b.

### *In situ* conductivity studies during nanocomposite synthesis

**Fig. 4:**
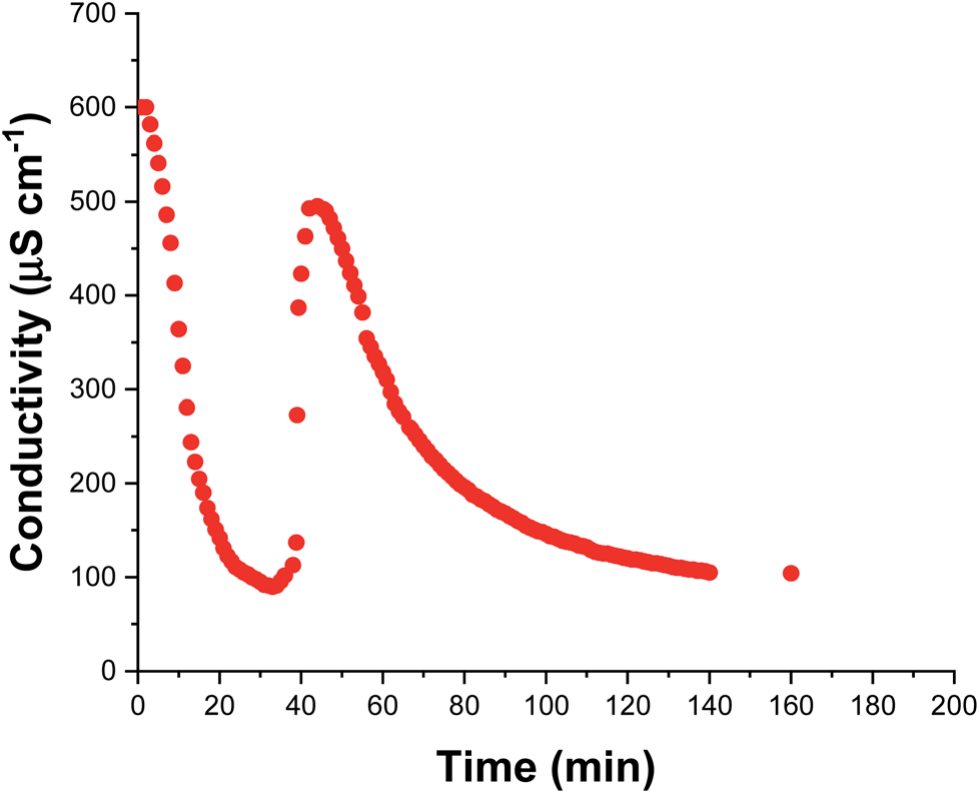
*In situ* solution conductivity measurements recorded during the aqueous emulsion polymerization of TFEMA in the presence of glycerol-functionalized silica nanoparticles at 60 °C targeting 10% w/w solids.

The solution conductivity can be monitored *in situ* providing valuable information during aqueous emulsion polymerization.^48^ This is particularly true when in the presence of surfactant as the solution conductivity depends mainly on the concentration of free surfactant dissolved within the aqueous continuous phase. During polymerization, this concentration changes as surfactant molecules adsorb to growing latex nanoparticles (and desorb from shrinking monomer droplets) which consequently alters the solution conductivity. Thus, monitoring the solution conductivity during polymerization can provide information regarding the underlying mechanism of polymerization.^48,49^ We recently reported that monitoring the solution conductivity *in situ* during the aqueous emulsion polymerization of TFEMA in the presence of SDS surfactant led to useful physical insights.^37^ In the present study, we conduct an aqueous emulsion polymerization using the same monomer (TFEMA), but in the presence of glycerol-functionalized silica nanoparticles^30^ rather than SDS surfactant. The glycerol groups replace some but not all of the silanol surface groups on the silica nanoparticles; ionization of the remaining silanol groups confers anionic surface charge, which contributes to the solution conductivity. Fig. 4 shows *in situ* solution conductivity data recorded during the synthesis of PTFEMA latex particles under the same conditions employed for the kinetic study shown in Fig. 2. A significant reduction in the solution conductivity from ∼600 to ∼85 μS cm^−1^ is observed over the first 35 min of the TFEMA polymerization, followed by a subsequent increase up to ∼500 μS cm^−1^ after 44 min. Interestingly, DLS measurements made during the equivalent kinetic study (Fig. 2b) indicate a change in the rate of particle growth after 35 min. The μm-sized TFEMA monomer droplets are stabilized by adsorbed silica nanoparticles. Thus such monomer droplets were postulated to disappear within 35 min, which would release the adsorbed anionic silica nanoparticles back into the continuous phase and hence account for the observed rapid increase in conductivity. Given the μm size range of the TFEMA monomer droplets, optical microscopy can be used to monitor their size during the TFEMA polymerization. Fig. S11† shows the evolution of monomer droplet size during polymerization: monomer droplets are clearly present at 30 min but barely visible after 40 min. This suggests that the observed increase in solution conductivity over this timescale does indeed correspond to the disappearance of the monomer droplets. Given that TFEMA is a good solvent for PTFEMA, this results in rapid diffusion of monomer into the nanocomposite particles, which accounts for the enhanced rate of polymerization that is observed after 40 min (see Fig. 2b). It is also noteworthy that Fig. S11† confirms the presence of very large monomer droplets (>50 μm diameter) at the beginning of the polymerization. After 45 min, the solution conductivity decreases steadily until reaching a constant value of ∼100 μS cm^−1^ after 140 min. This reduction in conductivity occurs because anionic silica nanoparticles continue to adsorb at the surface of the growing PTFEMA latex particles until a complete silica shell is formed. Furthermore, this 140 min timescale for the TFEMA polymerization is consistent with that indicated by both ^1^H NMR kinetics and DLS studies (Fig. 2).

### Time-resolved SAXS studies during nanocomposite formation

The electron density of silica is significantly higher than PTFEMA, despite the latter's semi-fluorinated nature. Thus, the X-ray scattering in the time-resolved SAXS experiments is dominated by the silica nanoparticles, which makes this technique particularly well-suited to monitoring the spatial location of this component within the growing nanocomposite particles. For example, an alternative route to similar core–shell nanocomposite particles involves the physical adsorption of a monolayer of silica nanoparticles onto pre-formed sterically-stabilized latex particles.^50,51^ This approach was reported by Balmer *et al.*,^52^ who subsequently used SAXS to confirm the redistribution of weakly adsorbed silica nanoparticles when poly(2-vinylpyridine)-silica nanocomposite particles were ‘challenged’ by addition of bare poly(2-vinylpyridine) latex particles.^53^ Moreover, time-resolved SAXS experiments indicated that such silica redistribution occurred within a few seconds for a dilute dispersion of a binary mixture of nanocomposite and latex particles at ambient temperature.^53^ There have been several postmortem SAXS studies of core–shell nanocomposite particles^13,53–56^ but to the best of our knowledge, the present study is the first time-resolved SAXS experiments to be conducted during *in situ* polymerization. In principle, this approach should provide new insights into the mechanism of nanocomposite formation in terms of both particle nucleation and subsequent growth. The stirrable reaction cell shown in Fig. 1 has been recently used to conduct *in situ* SAXS experiments during RAFT aqueous emulsion polymerization,^26^ RAFT aqueous dispersion polymerization^57^ and also conventional aqueous emulsion polymerization.^37^ Herein we utilize the same experimental set-up to monitor the formation of colloidal nanocomposite particles. Importantly, the sample volume of this stirrable reaction cell is around 2.0 mL, which is sufficient to enable postmortem characterization of the resulting PTFEMA/silica nanocomposite particles. A synchrotron X-ray source is essential to provide sufficient temporal resolution to monitor the relatively fast kinetics of the *in situ* TFEMA polymerization (Fig. 2). This enables many good-quality SAXS patterns to be recorded within short time scales. This enables both nucleation and the subsequent evolution in particle growth to be studied, as well as characterization of the final nanocomposite particles.

**Fig. 5:**
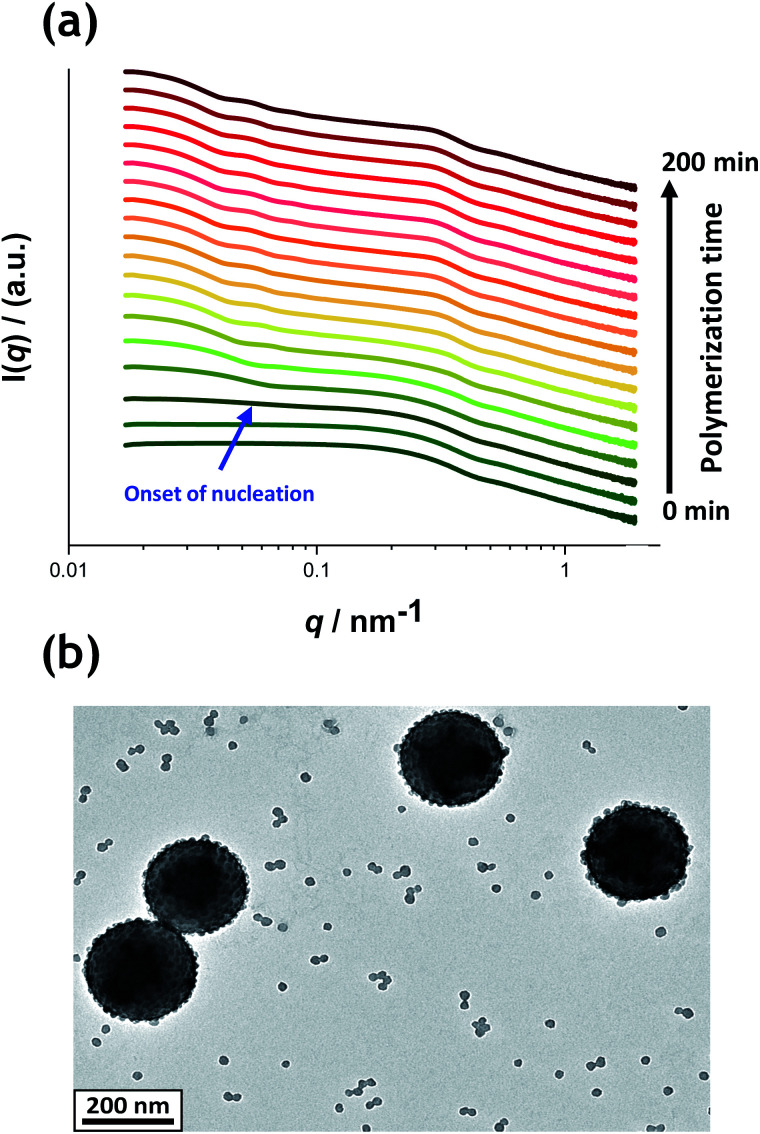
(a) SAXS patterns recorded *in situ* during the aqueous emulsion polymerization of TFEMA in the presence of a 19 nm diameter glycerol-functionalized silica sol (Bindzil CC401) using a cationic azo initiator at 60 °C when targeting 10% w/w solids. The onset of particle nucleation is indicated by the blue arrow. Scattering patterns are scaled by an arbitrary factor to avoid overlap and improve clarity. (b) Postmortem TEM image of the final PTFEMA/silica nanoparticles showing well-defined core–shell nanocomposites.

**Fig. 6:**
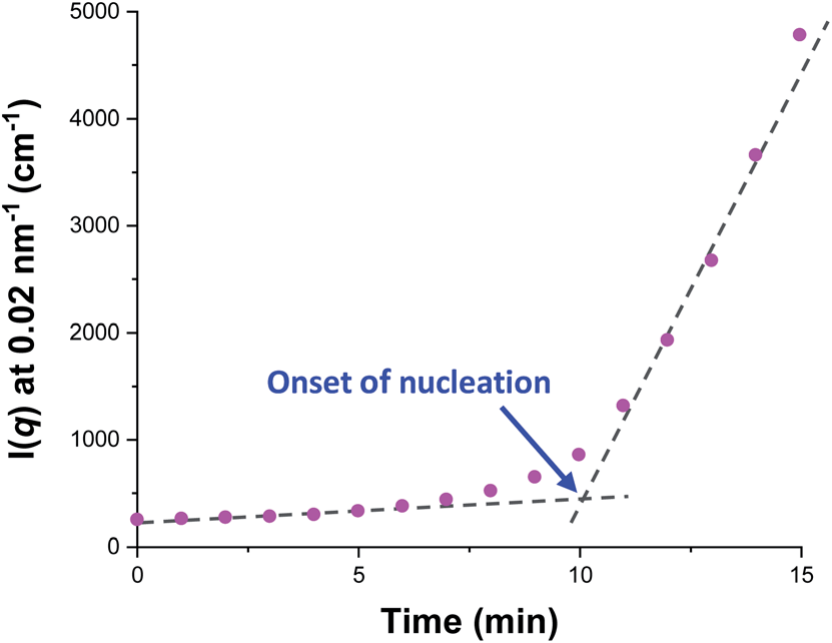
Evolution in *I*(*q*) recorded during the time-resolved SAXS experiment at an arbitrary *q* value of 0.02 nm^−1^ for the first 15 min of the aqueous emulsion polymerization of TFEMA in the presence of a 19 nm glycerol-functionalized silica sol using a cationic azo initiator at 60 °C. The onset of particle nucleation is highlighted by the blue arrow.

Fig. 5a shows the X-ray scattering intensity, *I*(*q*), plotted as a function of the scattering vector length, *q* [*q* = (4π sin *θ*)/*λ* where *λ* is the wavelength of X-ray radiation and *θ* is half of the scattering angle], for selected SAXS patterns recorded *in situ* during the aqueous emulsion polymerization of TFEMA in the presence of the glycerol-functionalized silica sol at 60 °C when targeting 10% w/w solids. During the first few minutes of polymerization, the scattering patterns mainly correspond to the high-contrast spherical silica nanoparticles. It should be noted that the monomer is present in the initial emulsion mixture as large droplets of approximately 50 μm in diameter (Fig. S11†), which are too large to be detectable by SAXS within the accessible *q*-range. The onset of polymer particle nucleation should lead to an increase in *I*(*q*) at low *q* because this parameter is proportional to the volume of the scattering object. Fig. 6 shows the variation in *I*(*q*) (recorded at an arbitrary *q* value of 0.02 nm^−1^) over time during the first 15 min of the TFEMA polymerization. The upturn in scattering intensity observed after approximately 10 min indicates the onset of nucleation (also highlighted in Fig. 5 by the blue arrow). Further scattering patterns recorded during the first 15 min of the polymerization are shown in Fig. S12 in the ESI.† The observed upturn in scattering owing to the formation of the nascent polymer/silica aggregates is highlighted in this additional plot. This agrees well with the nucleation event observed after around 13 min indicated by the DLS data (Fig. 2b) and the corresponding TEM images recorded for the equivalent laboratory-scale synthesis (Fig. 3). To a good approximation, the first scattering pattern recorded at 1 min corresponds to free silica nanoparticles. Thus, subtracting this scattering pattern from consecutive patterns recorded prior to nucleation (0–15 min) highlights the formation of PTFEMA nuclei, see Fig. S13.† The scattering pattern recorded after 8 min can be satisfactorily fitted using a simple sphere model, which indicates a volume-average particle diameter of 63 nm, see Fig. S13c.† This is consistent with TEM images recorded after 10 min (see Fig. 3b), which indicate the formation of nascent particles with a number-average particle diameter of 59 nm. After 11 min, over-subtraction of the scattering patterns at approximately *q* = 0.02 Å^−1^ leads to an apparent local minimum (see Fig. S13b†). This feature is the result of a structure factor peak originating from silica nanoparticles packed within the nascent polymer/silica aggregates. Clearly, the lower volume fraction of free silica nanoparticles means that it is no longer valid to use the initial scattering pattern (recorded after 1 min) for background subtraction. Hence this provides further evidence for nucleation occurring between approximately 10–15 min. The TFEMA polymerization was judged to be complete when no further change in the scattering pattern was discernible.^35^ This corresponded to a reaction time of approximately 150 min (see Fig. S3†), which agrees reasonably well with the time scale of 140 min indicated for the equivalent laboratory-scale synthesis using ^1^H NMR spectroscopy (see Fig. 2a). In prior time-resolved SAXS measurements during PISA syntheses, a significant rate enhancement was observed that was attributed to additional radicals generated by the high-flux X-ray beam.^35,36^ Interestingly, there is little or no evidence for an enhanced rate of polymerization in the present study, which seems to be a fairly general observation for aqueous formulations.^35,37,57^ This is fortunate, because it means that the kinetics of polymerization determined by ^1^H NMR studies of the laboratory-scale synthesis can be used to analyse the scattering patterns recorded during the time-resolved SAXS experiments.

^1^H NMR analysis of the quenched reaction mixture retrieved from the stirrable reaction cell used for the SAXS measurements indicated a final TFEMA conversion of 96% while DLS studies indicated a volume-average particle diameter of 234 nm (DLS polydispersity = 0.04). Such postmortem data are consistent with those obtained for the equivalent laboratory-scale synthesis shown in Fig. 2 (*i.e.* 96% TFEMA conversion, with a final volume-average particle diameter of 244 nm and a DLS polydispersity of 0.03). Furthermore, TEM analysis confirms the formation of well-defined core–shell PTFEMA/silica nanocomposite particles with a number-average particle diameter of approximately 215 nm (see Fig. 5b). The variation in contrast for the nanocomposite particles indicated by TEM studies in Fig. 3b–d and 5b simply reflects the significantly greater particle volume in the latter case, which attenuates the high-energy electron beam more effectively.

### Three-population scattering model used for SAXS analysis

SAXS has been previously used to characterize core–shell particles comprising either organic^58–60^ or inorganic shells.^53,61,62^ To further analyse the SAXS patterns shown in Fig. 5, a suitable scattering model is required. Previously, Balmer *et al.* employed a two-population model to analyse core–shell particles composed of a silica shell and either poly(2-vinylpyridine) or polystyrene cores.^53,55,56^ This two-population approach was required to account for (i) the particulate nature of the silica shell and (ii) repulsive interactions between neighbouring silica nanoparticles. However, Balmer *et al.* monitored the physical adsorption of silica nanoparticles onto pre-formed sterically-stabilized latex particles by measuring the evolution of the effective shell thickness formed by the silica nanoparticles. It was assumed that the averaged scattering length density of the shell was constant over the period of absorption, and no differentiation was made between free silica nanoparticles and adsorbed silica nanoparticles in the model.^56^ In contrast, the evolution of nanocomposite formation is monitored during polymerization by *in situ* SAXS in the current study. Therefore, a more sophisticated scattering model is required to describe the evolving structure of the nanocomposite particles and the other components present in the system (see ESI†). In order to obtain satisfactory fits to the scattering patterns recorded during the TFEMA polymerization, a third population had to be included in the model to account for the scattering contribution from the variable proportion of free (non-adsorbed) silica nanoparticles. Thus, the first population in eqn (S2)† (*i* = 1) describes the core–shell structure of the nanocomposite particles using a suitable spherical form factor and a hard-sphere structure factor that describes interparticle-correlations between nanocomposite particles (eqn (S3)–(S12)†). The second population (*i* = 2) describes the particulate nature of the silica shell using a spherical form factor and a hard-sphere structure factor that accounts for interparticle correlations between silica nanoparticles within the shell (eqn (S13)–(S15)†). The third population (*i* = 3) uses a spherical form factor (eqn (S16)–(S18)†) and accounts for the gradual reduction in concentration of the free silica nanoparticles present in the aqueous continuous phase. The Irena SAS macro^63^ for IgorPro was used to program the model and fit the SAXS patterns.

It has been assumed in the SAXS model that both the amount of silica nanoparticles (*i.e.* their total volume concentration corresponding to the second and third populations), and total mass of the monomer and polymer remain constant over the reaction course (eqn (S5), (S9), (S10), (S15), and (S18)† respectively). Prior to analysing the time-resolved SAXS data recorded during the nanocomposite particle synthesis (Fig. 5a), structural parameters for the silica nanoparticles alone were determined using the first frame of the SAXS patterns where the scattering signal is dominated by the silica particles. A satisfactory fit to this scattering pattern was obtained by assuming that only population 3 (free silica) is present in the sample (Fig. S4†). A volume-average particle diameter of 19.6 nm was determined (Table S1†), which is consistent with the manufacturer's specification of 19 nm. The final frame recorded during the time-resolved SAXS experiment (after approximately 180 min) was used to determine the relative volume fraction of polymer and, subsequently, the associated relative volume fraction of the monomer (Fig. S5 and Table S1†). The monomer conversion determined by postmortem ^1^H NMR analysis (96%) was used as the reference point to normalize the SAXS data. Scattering length densities (SLDs) for the silica, water, TFEMA monomer and PTFEMA homopolymer remained fixed within the scattering model and were calculated based on the respective chemical compositions and the densities of each component at the reaction temperature of 60 °C, see Table S1.† TFEMA monomer has a relatively low aqueous solubility (2.9 g dm^−3^ at 20 °C) so it mainly resides within μm-sized emulsion droplets (see Fig. S11†). Thus the SLD of the aqueous continuous phase was assumed to be that of water.

The time-resolved scattering patterns shown in Fig. 5a were analysed in reverse chronological order starting from the final frame by imposing the various constraints of the model and the known constant parameters (Table S1†). The three-population scattering model provides satisfactory fits to the scattering patterns after the first 18 min of polymerization (Fig. S6†). According to DLS (Fig. 2b), TEM (Fig. 3) and preliminary SAXS analysis (Fig. 6), this time point is close to the suggested onset of nucleation (approx. 13 min). It is reasonable to expect that the initial nascent particles possess somewhat ill-defined morphologies.^31^ Indeed, a well-defined core–shell morphology is not obtained until approximately 5 min after nucleation. Thus satisfactory data fits to the SAXS patterns can only be obtained for a sub-set of the data when employing such a scattering model. During the synthesis of these nanocomposite particles, the silica component is present in three forms: (i) initially, as freely diffusing nanoparticles within the aqueous continuous phase, then (ii) randomly adsorbed onto the growing PTFEMA nuclei at well below monolayer coverage, and finally (iii) as an increasingly well-defined contiguous shell surrounding the PTFEMA latex cores. Because not all of the initial silica nanoparticles are incorporated into the nanocomposite particles, there is always a background of free (non-adsorbed) silica nanoparticles at any given time. Thus, a population balance constraint was incorporated within the scattering model to ensure that the overall mass fraction of silica nanoparticles remained constant throughout the synthesis.^64,65^ This approach enables the evolution of both the silica particle aggregation efficiency (*A*_e_) and the packing density within the core–shell particle shell (*ϕ*_silica_) to be calculated during the polymerization. Furthermore, the scattering model also enables the mean silica shell thickness (*S*_t_) (see eqn (S11)†) and the instantaneous TFEMA monomer conversion to be determined. Owing to the relatively high final concentration of nanocomposite particles (10% w/w solids), a hard-sphere structure factor is introduced to account for particle–particle interactions.^66^ Furthermore, the same hard-sphere structure factor is used to describe the interaction of silica particles within the densely-packed shell (the second population).^66^ Full details of this sophisticated scattering model are provided in the ESI† and a summary of the fixed and fitted variables are provided in Tables S1 and S2,† respectively.

### SAXS analysis during nanocomposite formation

**Fig. 7:**
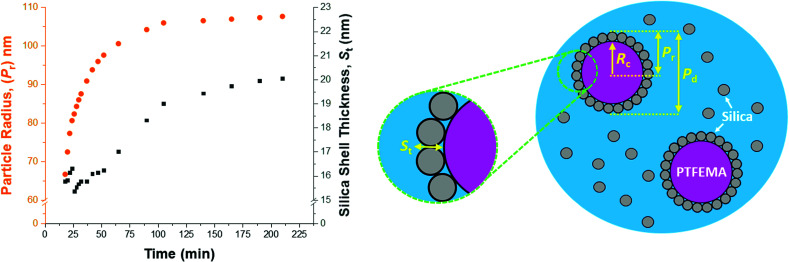
*In situ* SAXS studies conducted during the aqueous emulsion polymerization of TFEMA in the presence of a glycerol-functionalized silica sol at 60 °C to form PTFEMA/silica nanocomposite particles with a well-defined core–shell morphology. Data analysis enables the evolution in overall particle radius (*P*_r_), the core radius (*R*_c_) and the mean silica shell thickness (*S*_t_) (where *P*_r_ = *R*_c_ + *S*_t_) to be conveniently monitored.

In view of the ill-defined nature of the nascent polymer/silica aggregates, the three-population scattering model cannot be used to describe such transient species. Instead, only SAXS patterns recorded after nucleation (approximately 18 min) were fitted using the three-population model. Fig. 7 shows the evolution in overall particle radius (*P*_r_) and mean silica layer thickness (*S*_t_). The particle radius is calculated using *P*_r_ = *R*_c_ + *S*_t_, where *R*_c_ corresponds to the PTFEMA latex core radius, see Fig. 7 for a schematic representation. Hence the overall particle diameter, *P*_d_, is given by *P*_d_ = 2*P*_r_. Inspecting Fig. 7, the rate of particle growth is retarded significantly after 100 min. At this time point, a volume-average nanocomposite particle radius of approximately 108 nm (*P*_d_ = 216 nm) is observed, which is consistent with postmortem DLS and TEM data (*z*-average diameter = 234 nm and number-average diameter = 215 nm, respectively). Fig. 7 suggests that the nanocomposite particle radius changes by only approximately 1 nm during the last 100 min of the TFEMA polymerization. This minimal increase is the result of the continuous slow growth of the effective silica shell thickness (*S*_t_) rather than a subtle increase in the PTFEMA core radius (see Fig. 7). This suggests that silica nanoparticles within the shell undergo local rearrangement towards the end of the polymerization to achieve a higher packing efficiency. Based on the evolution of the overall nanocomposite particle radius, the TFEMA polymerization appears to be more or less complete within 140 min, which agrees well with the timescale indicated by ^1^H NMR studies of the equivalent laboratory-scale synthesis (Fig. 2a). Furthermore, the instantaneous TFEMA monomer conversion calculated using the population balance model also suggests that the polymerization is essentially complete within 140 min (Fig. S7a†). It is also possible to evaluate the volume fraction of monomer within the core (Fig. S7b†). During the first 50 min of polymerization, this parameter increases monotonically because monomer continues to diffuse from the giant monomer droplets to the growing latex particles during the early stages of polymerization. After 50 min, the volume fraction of TFEMA monomer within the growing particle cores steadily decreases until it becomes constant after 140 min. The gradual reduction in the monomer concentration within the growing particles shown in Fig. S7b† is consistent with the *in situ* conductivity data shown in Fig. 4, which suggests that the interval II/III transition occurs at approximately 50 min.^37^ During the latter stages of the TFEMA polymerization, the large monomer droplet reservoirs disappear and the remaining TFEMA monomer solely resides within the PTFEMA latex particles. This interpretation is supported by optical microscopy studies of the monomer droplets (Fig. S11†). Hence the volume fraction of unreacted monomer within the growing particle cores steadily decreases after the time point for the interval II/III boundary, which corresponds to monomer-starved conditions, as shown in Fig. S7b.†

According to Fig. S8,† DLS studies performed on aliquots extracted from the laboratory-scale synthesis (Fig. 2b) indicate a significantly faster rate of particle growth during the first 30 min of polymerization compared to that determined from *in situ* SAXS studies. However, DLS analysis of the laboratory-scale synthesis (Fig. 2b) and the corresponding *in situ* SAXS studies (Fig. 7) both indicate a period of rapid growth that continues up to approximately 28 min. This is then followed by a slower growth rate up to approximately 90 min. After this point, there is a subtle reduction in DLS particle diameter (presumably owing to shrinkage of the monomer-swollen particles as the low-density TFEMA is converted into high-density PTFEMA) until a constant final particle size is observed after 140 min.

From the evolution in *S*_t_ shown in Fig. 7, the initial effective shell thickness after nucleation is approximately 17 nm. Given that the volume-average diameter of an individual silica nanoparticle is 19 nm, a silica shell thickness of 17 nm observed during the early stages of polymerization implies either an incomplete shell (*i.e.* submonolayer coverage) or perhaps partial embedding of the silica nanoparticles within the PTFEMA cores. Indeed, Schmid *et al.* reported that silica nanoparticles become embedded within polystyrene cores during the synthesis of closely related colloidal nanocomposite particles.^9^ The apparent silica shell thicknesses determined when using the three-population scattering model to study the early stages of the TFEMA polymerization (*i.e.* immediately after nucleation) should be treated with extreme caution. This is because the nascent particles are unlikely to have acquired the core–shell morphology that is explicitly assumed when fitting the SAXS patterns. This is corroborated by TEM studies of aliquots extracted during the laboratory-scale synthesis, which do not provide any evidence for well-defined silica shells being formed on this timescale (Fig. 3). It is also possible to estimate the packing density of silica nanoparticles within the shell (*ϕ*_silica_, eqn (S5)†), see Fig. S10.† Interestingly, *ϕ*_silica_ is highest during the early stages of the TFEMA polymerization, which is also the case for the nanocomposite shell SLD (Fig. S9†). Given that *S*_t_ reaches its minimum value immediately after nucleation, this suggests a relatively dense but rather ill-defined silica shell. Although this is physically possible, it seems rather unlikely: the relatively small *S*_t_ combined with a high effective shell SLD (Fig. S9†) is most probably an artefact arising from the inherent limitations of the scattering model. During the TFEMA polymerization, the mean silica shell thickness increases up to around 20 nm which is close to the volume-average diameter of an individual silica nanoparticle (19 nm). This strongly suggests that the shell comprises a monolayer of adsorbed silica nanoparticles. Furthermore, a silica packing density (*ϕ*_silica_) of 47% is observed within 100 min of the polymerization (Fig. S10†). This experimental value is in good agreement with Monte Carlo simulations, which suggest that the maximum packing density for randomly-packed small spheres on the surface of a larger central sphere is about 45%.^55^

**Fig. 8:**
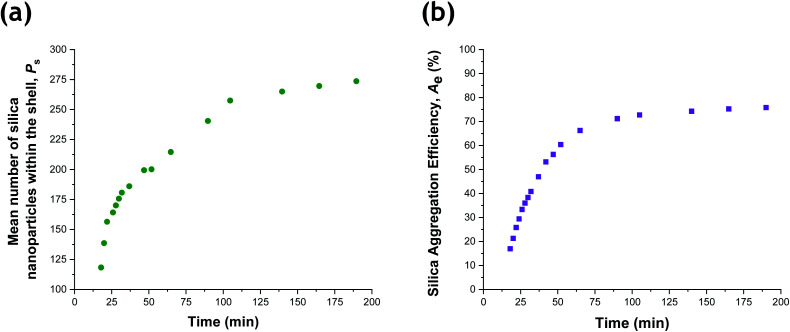
(a) Evolution in the number of silica nanoparticles within the shell, *P*_s_, and (b) the silica aggregation efficiency, *A*_e_, during the aqueous emulsion polymerization of TFEMA in the presence of a glycerol-functionalized silica sol at 60 °C.

Given that the silica volume fraction within the system necessarily remains constant, the silica nanoparticle aggregation efficiency, *A*_e_, can be estimated from the silica volume fraction within the shell. The former parameter should not be confused with the packing density, *ϕ*_silica_. The aggregation efficiency describes the proportion of the original silica nanoparticles that is incorporated within the shell, whereas *ϕ*_silica_ describes how efficiently silica nanoparticles can be packed around a large central PTFEMA sphere. Furthermore, it is also possible to calculate the mean number of silica nanoparticles within the shell (*P*_s_), see eqn (S20).†Fig. 8 shows the evolution in *P*_s_ and *A*_e_ during the TFEMA polymerization. Inspecting Fig. 8a, there is a sharp increase in *P*_s_ during the first 30 min of polymerization, after which *P*_s_ increases at a slower rate up to 100 min. At this time point, the rate of increase in *P*_s_ is further retarded until a limiting value of 274 silica nanoparticles is attained after 190 min. Interestingly, this evolution in *P*_s_ is similar to the evolution in overall particle radius, *P*_r_, shown in Fig. 7. This is physically reasonable because the number of adsorbed silica nanoparticles is governed primarily by the size of the PTFEMA cores. Thus, a commensurate increase in *P*_s_ should be observed as the TFEMA polymerization proceeds and the PTFEMA cores grow larger. This is also reflected in Fig. 8b, which shows the evolution in *A*_e_ during the polymerization. SAXS analysis indicates that a silica aggregation efficiency of approximately 75% is achieved by the end of the polymerization.

The silica aggregation efficiency can be independently determined by thermogravimetric analysis (TGA).^12,38,67^ First, the excess (non-adsorbed) silica nanoparticles are removed from the PTFEMA/silica nanocomposite particles *via* multiple centrifugation–redispersion cycles (see Experimental section 1.1 in the ESI†). The resulting purified PTFEMA/silica nanocomposite particles are dried to constant mass and then heated up to 800 °C in air to ensure complete pyrolysis of the organic component, leaving only the thermally stable silica nanoparticles as a residue. From the silica mass fraction determined by TGA, the silica aggregation efficiency can be calculated (see ESI,† section 2.1). Dried silica nanoparticles alone lose 3.8% mass on heating to 800 °C in air (see Fig. S1a†). This is attributed to (i) loss of surface moisture and (ii) pyrolysis of the surface glycerol groups. This mass loss is taken into account when calculating the silica content of the purified nanocomposite particles (see section 2.1 in the ESI†). Analysis of the purified PTFEMA/silica nanocomposite particles retrieved from the stirrable reaction cell after the *in situ* SAXS experiment indicated a silica aggregation efficiency of 68%. Bearing in mind the various experimental errors and uncertainties, this is in satisfactory agreement with the silica aggregation efficiency of 75% calculated from the SAXS data. Furthermore, the silica packing efficiency around the PTFEMA cores enables calculation of the mean silica content per nanocomposite particle,^55^ which is 26% by mass. For comparison, TGA studies indicate a residual silica mass of approximately 23%. Clearly, these data are consistent with a well-defined core–shell morphology with little or no silica nanoparticles present within the PTFEMA cores. Somewhat higher silica aggregation efficiencies of up to 95% have been reported for polystyrene/silica nanocomposites prepared by *in situ* polymerization in the presence of the same glycerol-functionalized silica nanoparticles.^12^ According to Schmid *et al.*,^9^ it may well be possible to achieve higher silica aggregation efficiencies for the present nanocomposite formulation by further optimizing the concentration of the silica nanoparticles and that of the cationic initiator, but this refinement is beyond the scope of the present study. Nevertheless, this new PTFEMA/silica nanocomposite formulation confirms that the judicious combination of a glycerol-functionalized silica sol with a suitable cationic azo initiator enables the synthesis of colloidally stable nanocomposite particles while achieving a reasonably high silica aggregation efficiency.

## Conclusions

The surfactant-free aqueous emulsion polymerization of TFEMA in the presence of a glycerol-functionalized silica sol using a cationic azo initiator at 60 °C leads to the formation of well-defined core–shell PTFEMA/silica nanocomposite particles. Using a stirrable reaction cell to perform time-resolved SAXS studies, we have monitored the *in situ* evolution from silica nanoparticles in co-existence with monomer droplets to micellar nucleation and subsequent particle growth. The cell volume is approximately 2.0 mL, which is sufficient to enable postmortem analysis of the final core–shell nanocomposite particles using ^1^H NMR spectroscopy, DLS, TEM and TGA.

Unlike previous *in situ* synchrotron SAXS studies,^35,36^ the high-flux X-ray radiation appears to have little or no effect on the rate of polymerization. This is fortunate, because it means that the kinetics of polymerization established for laboratory-scale syntheses can be applied to the *in situ* SAXS studies performed using the stirrable reaction cell. In particular, time-resolved SAXS measurements indicate that particle nucleation occurs within 10–15 min of polymerization. Once nucleation has occurred, nascent core–shell particles are observed by TEM and SAXS patterns can be satisfactorily fitted using a three-population scattering model for the growing core–shell particles that incorporates a population balance approach to account for both the particulate nature of the silica shell and also the non-adsorbed silica nanoparticles that remain within the aqueous continuous phase. This enables the nanocomposite particle diameter, silica shell thickness, mean number of silica nanoparticles within the shell, silica aggregation efficiency and packing density within the silica shell to be monitored during the TFEMA polymerization.

Immediately after the nucleation event, there is an initial period of rapid particle growth with a concomitant increase in the number of silica nanoparticles located within the shell. After approximately 60 min (50% TFEMA conversion) the rate of particle growth is reduced and the number of silica nanoparticles within the shell remains relatively constant. A final silica shell thickness of 20 nm is calculated, which is consistent with approximate monolayer coverage of the latex cores by the silica nanoparticles. ^1^H NMR spectroscopy studies indicate an overall TFEMA conversion of 96%, while SAXS analysis indicates a final volume-average core–shell particle diameter of 216 nm and a silica aggregation efficiency of approximately 75%, which are consistent with postmortem DLS, TEM and TGA studies. In summary, this time-resolved SAXS study has (i) shed new light on the mechanism of formation of polymer/silica nanocomposite particles and (ii) sets a new standard for their structural characterization that should inform the design of next-generation formulations for various commercial applications.

## Author contributions

A. Czajka conducted all experiments and analysed most of the data. G. Liao analysed the SAXS data using the population balance model. O. O. Mykhaylyk supervised G. Liao and co-developed the population balance model. S. P. Armes obtained the funding and supervised A. Czajka. The manuscript was co-written by all four authors.

## Conflicts of interest

There are no conflicts to declare.

## Supplementary Material

SC-012-D1SC03353K-s001
