# Supplementary material for: In situ small-angle X-ray scattering studies during the formation of polymer/silica nanocomposite particles in aqueous solution
Source: Chem Sci. 2021 Oct 20;12(42):14288–300. doi: 10.1039/d1sc03353k (PMC8565378; doi:10.1039/d1sc03353k)
Supplement: SC-012-D1SC03353K-s001 [file SC-012-D1SC03353K-s001.pdf]

Electronic Supplementary Material (ESI) for Chemical Science.
This journal is © The Royal Society of Chemistry 2021

Supporting Information for:

In situ Small-Angle X-ray Scattering Studies During the Formation of Polymer/Silica Nanocomposite Particles in Aqueous Solution

A. Czajka*, G. Liao, O. O. Mykhaylyk and S. P. Armes*

Table of Contents

1) Experimental Section S2
1.1) Polymer Characterization. S3

2) Supporting Analysis S6
2.1) Calculating Silica Aggregation Efficiency from TGA S6

3) Supporting Table and Figures S8
3.1) *Postmortem* TEM Images recorded for Nanocomposite Particles S8
3.2) Particle Nucleation S9
3.3) Fit to SAXS Pattern for Silica Nanoparticles S10
3.4) Fit to Final SAXS Pattern S11
3.5) Representative Fits S12
3.6) Conversion vs. Time Curve Determined from SAXS Fitting S13
3.7) Comparing Particle Size Determined by DLS and *in situ* SAXS S14
3.8) Silica shell SLD S15
3.9) Silica shell packing density, ϕ_{silica} S16
3.10) Optical Microscopy Images S17
3.11) Scattering Patterns during Polymerization S18
3.12) Scattering Patterns Prior to Nucleation S19

4) SAXS Model S20
4.1) Scattering Model for SAXS Analysis S20

1. Experimental Section

Materials

The following chemicals were used without further purification unless stated. 2,2,2-Trifluoroethyl methacrylate (TFEMA; Aldrich, 99%) was passed through a MEHQ inhibitor removal column prior to use. The Bindzil CC401 glycerol-modified aqueous silica sol (19 nm diameter; 40% w/w) was supplied by Nouryon (Bohus, Sweden). 2,2'-Azobis(2-isobutyramidine) dihydrochloride (AIBA; 97%), $MgSO_4$ (98%) and $CDCl_3$ (99.8%) were purchased from Sigma-Aldrich (UK). Deionized water was obtained from an Elga DV25 water purifier.

Laboratory-scale synthesis of PTFEMA/silica nanocomposite particles

AIBA initiator (74.8 mg; 1.0 mol % based on TFEMA), Bindzil CC401 silica sol (4.71 g of a 40% w/w dispersion, or 1.89 g dry silica) and deionized water (55.3 mL) were weighed into a 100 mL round-bottom flask containing a magnetic stirrer bar. The reaction mixture was adjusted to pH 8.9 by addition of 200 µL of a 0.1 M NaOH solution and then degassed with N_2 gas for approximately 30 min. After removing its MEHQ inhibitor, cold TFEMA was degassed separately using N_2 gas for 30 min with the aid of an ice bath. Degassed TFEMA (4.53 g) was then added to the reaction mixture and a 0.1 mL aliquot was immediately extracted for ^{1}H NMR spectroscopy analysis. The reaction solution was degassed for a further 5 min prior to immersion in a 60 ^{o}C oil bath and stirred magnetically at 800 rpm. The 'zero time' (t = 0 min) for this polymerization was arbitrarily taken to be the point when the degassed reaction solution was first immersed in the oil bath, rather than the time at which the reaction solution had reached this temperature. Aliquots were subsequently removed under N_2 via syringe at various time intervals for ^{1}H NMR, DLS and TEM analysis. Each 1.0 mL aliquot was quenched by cooling using an ice bath with concomitant exposure to air. For ^{1}H NMR analysis, 40 µL of each aliquot was diluted using $CDCl_3$ (650 µL). A small quantity of anhydrous $MgSO_4$ was added to remove water and each deuterated solution was then passed through a cotton wool-plugged pipette to remove any solids. For both TEM and DLS analysis, each aliquot was diluted fifty-fold using deionized water at 20 ^{o}C to produce 0.20% w/w dispersions.

In situ SAXS studies of nanocomposite particle formation using the stirrable reaction cell

AIBA initiator (3.0 mg; 1.0 mol % based on TFEMA), Bindzil CC401 silica sol (0.187 g of a 40% w/w dispersion, or 74.7 mg dry silica) and deionized water (2.21 mL) were weighed into a 14 mL sample vial. The reaction mixture was adjusted to pH 8.9 by addition of 10 µL of a 0.1 M NaOH solution and then degassed with N_2 gas for approximately 30 min. After removing its MEHQ inhibitor, cold TFEMA was degassed separately using N_2 gas for 30 min with the aid of

an ice bath. Degassed TFEMA (0.18 g) was added to the reaction mixture, which was then transferred via degassed syringe to the stirrable reaction cell (already containing a magnetic flea and equipped with a magnetic stirrer unit) which had been separately purged with N_2 gas for 20 min. This cell was then attached to the sample stage in I22 and aligned relative to the X-ray beam and TFEMA polymerization was initiated by using a water-circulating jacket to heat the cell up to 60 ^{o}C as the X-ray beam shutter was opened. The TFEMA polymerization was monitored until no further evolution in the 1D SAXS pattern was observed, at which point it was assumed that the reaction was complete.

In situ conductivity studies during the aqueous emulsion polymerization of TFEMA in the presence glycerol-functionalized silica nanoparticles

The experimental protocol employed for determining the solution conductivity during the synthesis of silica-stabilized PTFEMA latex particles was as follows. A 100 mL two-neck round-bottom flask was fitted with a Primo 5 conductivity probe. Bindzil CC401 (6.41 g of a 40% w/w dispersion, or 2.56 g dry silica) and deionized water (70 mL) were weighed into the 100 mL round-bottom flask. The reaction mixture was adjusted to pH 8.9 by adding 0.1 M NaOH solution (100 µL) via micropipette and then degassing with N_2 gas at 60 °C for approximately 30 min. AIBA initiator (0.102 g) and deionized water (5 mL) were weighed into a 14 mL sample vial and degassed with N_2 gas for approximately 30 min. After removing its MEHQ inhibitor, cold TFEMA was degassed separately using N_2 gas for 30 min with the aid of an ice bath. Degassed TFEMA (6.14 g) was added to the reaction mixture and allowed to stir at 500 rpm for 3 min. The degassed aqueous AIBA solution was then added to the reaction mixture. The ‘zero time’ (t = 0 min) for this polymerization was arbitrarily taken to be the point when the degassed initiator solution was added to the reaction mixture. Conductivity data from the Primo 5 were recorded using the camera facility on a mobile phone. ^{1}H NMR analysis indicated that a final TFEMA monomer conversion of 98% was achieved during this synthesis.

1.1 Polymer Characterization

^{1}H NMR Spectroscopy

All NMR spectra were recorded at 298 K using a 400 MHz Bruker Avance-400 spectrometer (64 scans averaged per spectrum).

Dynamic Light Scattering (DLS)

DLS studies were conducted on 0.20% w/w aqueous dispersions at 25 °C in disposable plastic cuvettes using a Malvern Zetasizer NanoZS instrument that detects back-scattered light at an angle of 173°. Intensity-average hydrodynamic diameters were calculated via the Stokes−Einstein equation using a non-negative least squares (NNLS) algorithm. All data were averaged over three consecutive runs. Intensity-average hydrodynamic diameters were converted into volume-average hydrodynamic diameters using the Malvern Zetasizer Software (Version 7.01).

Transmission Electron Microscopy (TEM)

Removed aliquots were diluted fifty-fold at 20 °C to generate 0.20% w/w dispersions. Copper/palladium TEM grids (Agar Scientific, UK) were surface-coated in-house to yield a thin film of amorphous carbon. The grids were then plasma glow-discharged for 30 s to create a hydrophilic surface. Individual samples (0.20% w/w, 5 µL) were adsorbed onto the freshly glow-discharged grids for 1 min and then blotted with filter paper to remove excess solution. To stain the aggregates, uranyl formate solution (0.75% w/v, 5 µL) was soaked on the sample-loaded grid for 20 s and then carefully blotted to remove excess stain. The grids were then dried using a vacuum hose. Imaging was performed on a Technai T12 Spirit instrument at 120 kV equipped with a Gatan 1 k CCD camera.

Thermogravimetric Analysis (TGA)

Thermogravimetric analyses were conducted using a Perkin-Elmer Pyris 1 TGA instrument. Excess non-adsorbed silica nanoparticles remaining after the TFEMA polymerization was removed via five centrifugation-redispersion cycles (5,000 rpm for 20 min), with each successive supernatant being carefully decanted and replaced with deionized water. TEM studies confirmed that excess silica nanoparticles were removed using this purification protocol. Higher centrifugation rates (> 8,000 rpm) and longer times (> 1 h) were avoided since these resulted in partial sedimentation of the non-adsorbed silica nanoparticles and also hindered redispersion of the sedimented nanocomposite particles. Purified nanocomposite dispersions were dried in an oven at 75 °C for 24 h and freeze-dried overnight remove

unreacted TFEMA and water. Using the Perkin-Elmer TGA instrument, dried samples were heated in air up to 800 °C at a heating rate of 10 °C min^{-1}. The observed mass loss was attributed to the quantitative degradation of PTFEMA, with the remaining white incombustible residues being assumed to be that of pure silica (SiO_2). The silica aggregation efficiency can be calculated from the residual mass, see Section 2.3.

Optical Microscopy

Aliquots were extracted from the reaction mixture at 60 °C and the TFEMA polymerization was quenched by cooling to 20 °C with concomitant exposure to air. Optical microscopy images were recorded immediately at 20 °C using a Motic DMBA300 digital biological microscope equipped with a built-in camera and Motic Images Plus 2.0 ML software.

Small-Angle X-ray Scattering (SAXS)

SAXS patterns were recorded at a synchrotron facility (station I22 at Diamond Light Source, Didcot, Oxfordshire, UK). A monochromatic X-ray beam (λ = 0.124 nm) and a 2D Pilatus 2M pixel detector (Dectris, Baden, Switzerland), were used for these experiments. A q range of 0.02−2.00 nm^{-1} was used for measurements, where $q = (4\pi \sin \theta)/\lambda$, corresponds to the modulus of the scattering vector and θ is half of the scattering angle. For these time-resolved measurements, a custom-designed SAXS cell was used as the sample holder, see Figure 1 in the main text. SAXS patterns were recorded every 60 seconds for 60 min and every 150 seconds thereafter until no further change in the SAXS patterns could be observed. X-ray scattering data were reduced (integrated, normalized, and background-subtracted) using Dawn software supplied by Diamond Light Source. The scattering intensity of water was used for absolute scale calibration of the X-ray scattering patterns. Irena SAS macros for Igor Pro were utilized for modelling and further SAXS analysis.

2. Supporting Analysis

2.1 Calculating Silica Aggregation Efficiency from TGA

The silica nanoparticles used in this work lose 3.8% mass on heating to 800 °C in air, see Figure S1a. This is attributed to a (i) surface moisture and (ii) pyrolysis of surface glycerol groups, which are present for this particular commercial grade. This mass loss must be taken into account when calculating the silica content of the nanocomposite particles. In order to account for this, the residual mass as determined by TGA for the purified nanocomposite particles must be divided by the silica weight remaining (96.2) divided by 100:

$$s = \frac{s_{\mathrm{nanoparticles}}}{s_{\mathrm{silica}}/100}$$

where $s_{\mathrm{nanoparticles}}$ and s_{silica} are the mass fractions of the purified nanoparticles and silica particles determined by TGA (see Figure S1). Figure S1b shows the TGA curve recorded for the purified nanocomposite particles retrieved from the stirrable reaction cell after the *in situ* SAXS experiment (i.e., after the excess non-adsorbed silica nanoparticles were removed by five centrifugation-redispersion cycles). A residual mass of 21.9% was determined by TGA for the purified nanocomposite particles , thus *s* = 22.8%.

With the modified silica mass fraction of the nanocomposite particles calculated, the silica aggregation efficiency can be determined using:

$$x = \frac{s m_{\mathrm{monomer}} c}{(1-s) m_{\mathrm{silica}}}$$

where m_{monomer} and m_{silica} are the initial masses of monomer and silica respectively, c is the TFEMA conversion, and s is the modified silica mass fraction of the nanocomposite particles. At the end of the TFEMA polymerization, the silica aggregation efficiency can be calculated as follows:

$$x = \frac{0.228 * 0.18 * 0.96}{(1-0.228) * 0.0747\ \mathrm{g}} = 68.3\%$$

Parameter	Value
m_{monomer}	0.18 g
m_{silica}	0.0747 g
c	0.96 (96%)
s	0.228 (22.8%)

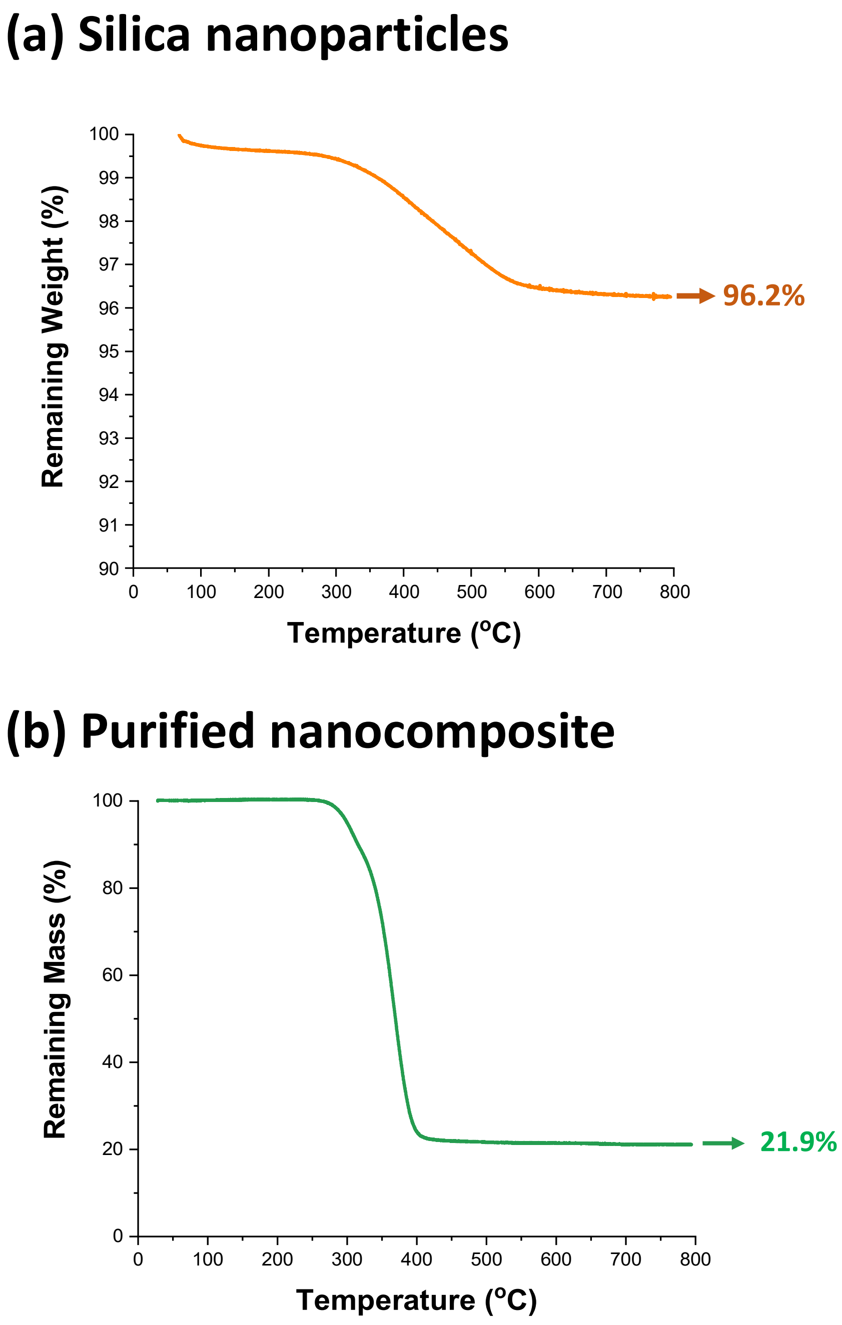

Figure S1. TGA curve recorded for (a) the silica nanoparticles after being freeze-dried overnight (b) the purified nanocomposite particles retrieved from the stirrable reaction cell after the *in situ* SAXS experiment (i.e., after the excess non-adsorbed silica nanoparticles were removed by five centrifugation-redispersion cycles prior to drying). To pyrolyze the components, samples were heated up to 800 °C in air at a heating rate of 10 °C min^{-1}.

3. Supporting Figures

3.1 *Postmortem* TEM images recorded for Nanocomposite Particles

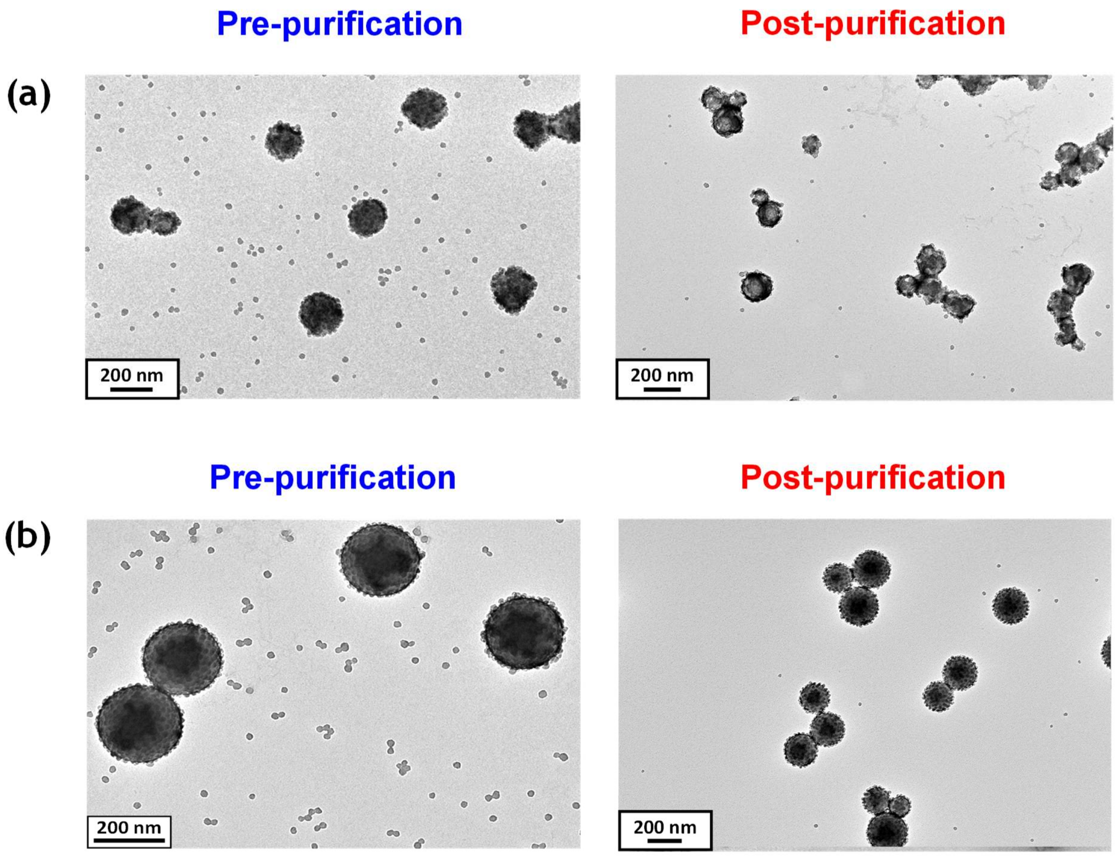

Figure S2. *Postmortem* TEM images recorded for PTFEMA/silica nanocomposite particles prepared by aqueous emulsion polymerization of TFEMA in the presence of glycerol-functionalized silica nanoparticles using a cationic azo (AIBA) initiator at 60 °C: (a) laboratory-scale synthesis and (b) stirrable reaction cell used for the *in situ* SAXS experiments. Purification refers to the five centrifugation-redispersion cycles required to remove excess non-adsorbed silica nanoparticles (see Section 1.1).

3.2 Particle Nucleation

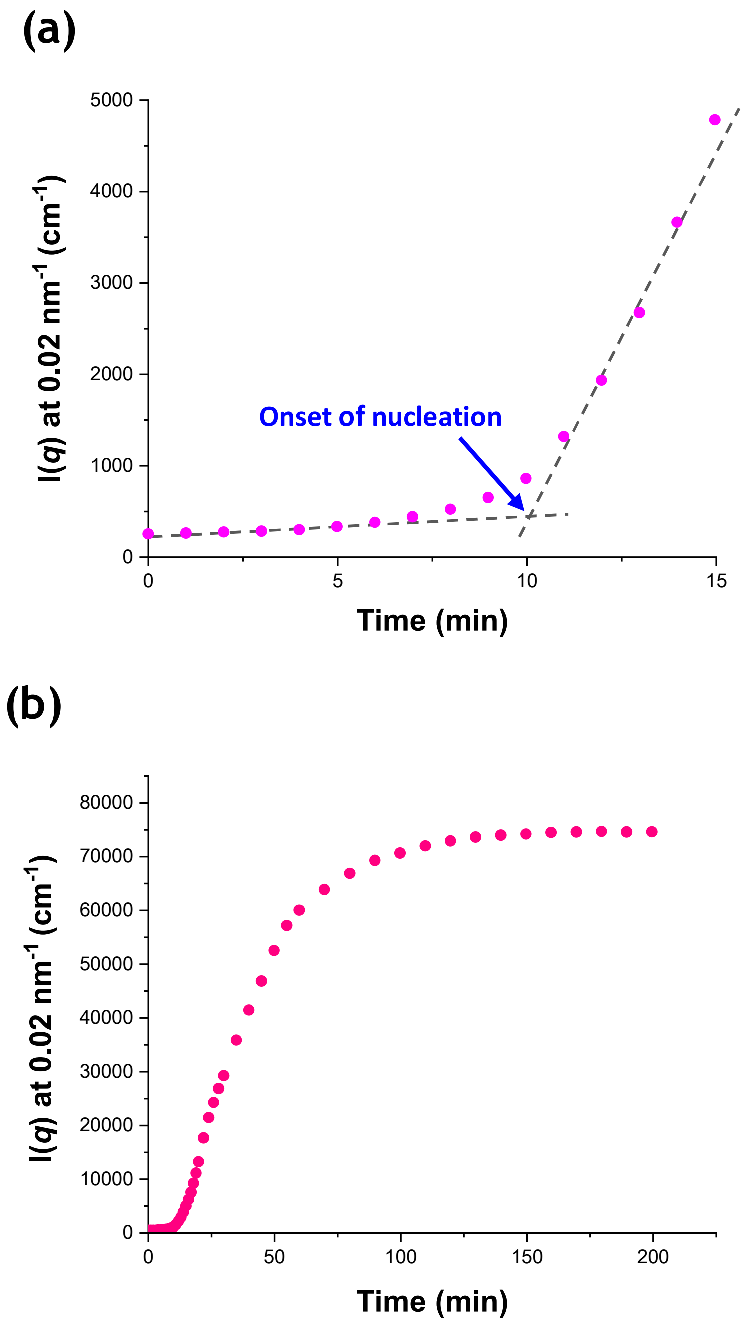

Figure S3. Scattered X-ray intensity I(q) at an arbitrary q value of 0.02 nm^{-1} recorded for the aqueous emulsion polymerization of TFEMA at 60 ˚C in the presence of glycerol-functionalized silica nanoparticles using a cationic azo initiator: (a) during the first 15 min of TFEMA polymerization, whereby the upturn in intensity at approximately 10 min corresponds to the onset of particle nucleation; (b) throughout the course of the TFEMA polymerization, whereby no further change in I(q) recorded at an arbitrary q value of 0.02 nm^{-1} indicates the end of this reaction.

3.3 Fits to SAXS Pattern for Silica Nanoparticles

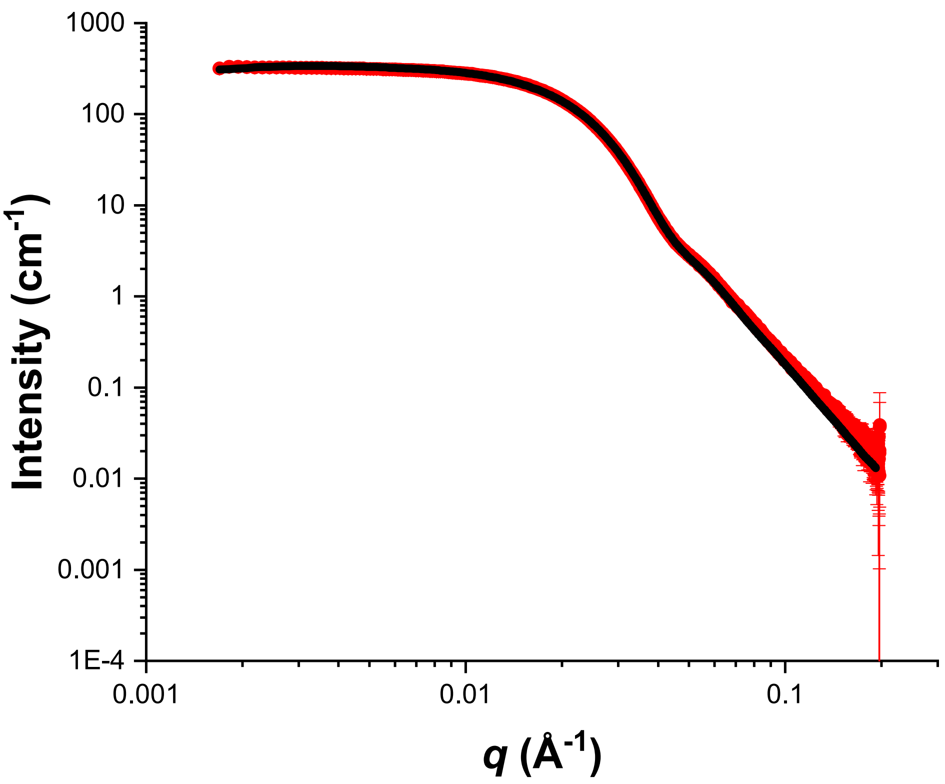

Figure S4. First frame (see red circles) recorded during the aqueous emulsion polymerization of TFEMA in the presence of glycerol-functionalized silica nanoparticles at 60 °C targeting 10% w/w solids. The data fit obtained when using a spherical form factor (population 3 of the three-population model) is shown as a black line. The resulting calculated parameters are summarized in Table S1.

3.4 Fit to Final SAXS Pattern

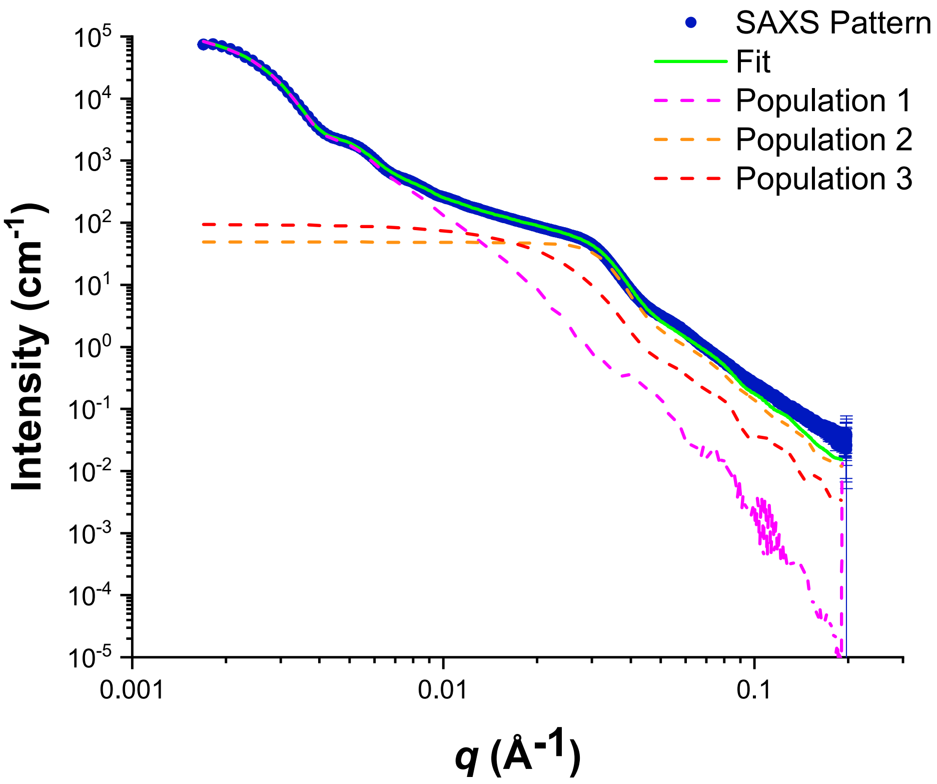

Figure S5. Breakdown of the fit (green line) to the final scattering pattern (blue circles) showing the three individual populations as pink, orange and red dashed lines for the first, second and third populations, respectively.

3.5 Representative SAXS Data Fits

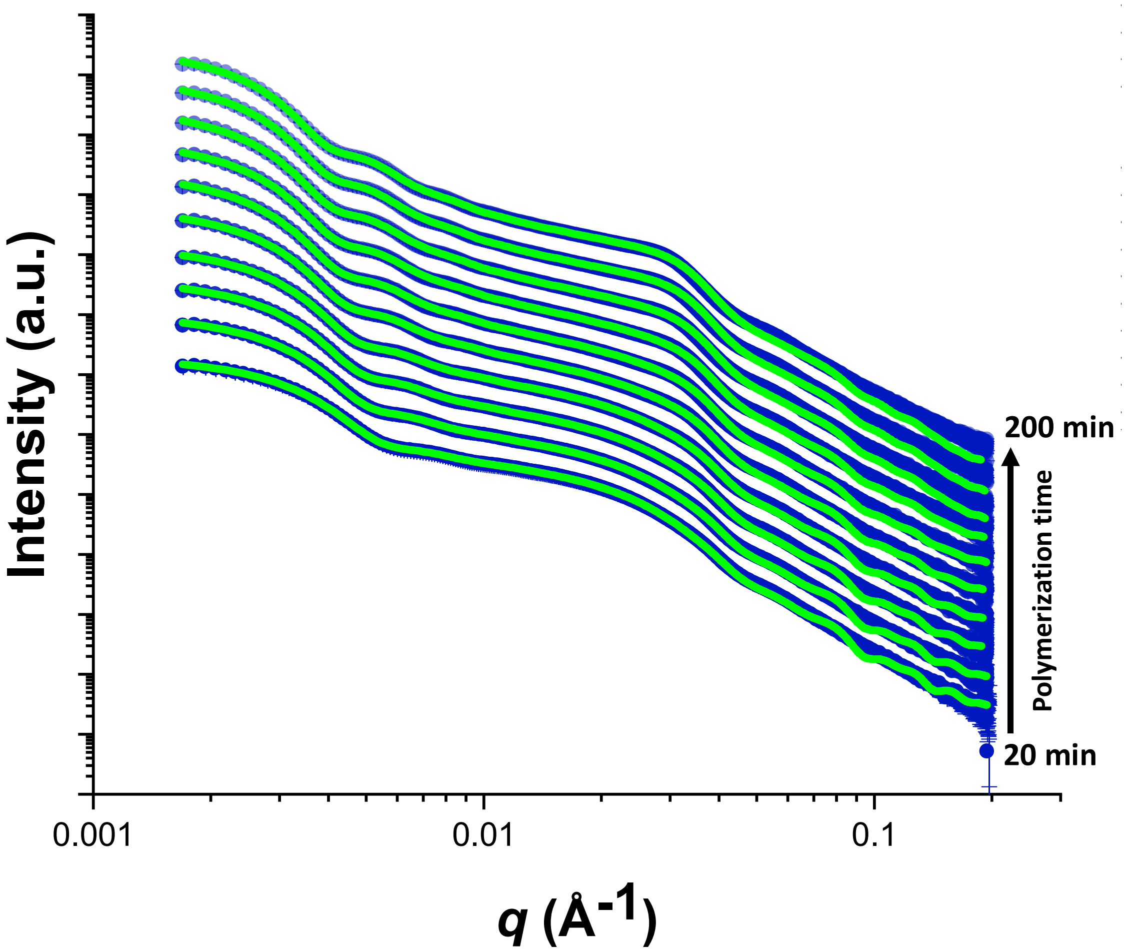

Figure S6. Selected SAXS patterns (blue circles) with corresponding fits (green lines) obtained using the three-population model. Data have been arbitrarily scaled to reduce overlap between scattering patterns and improve clarity.

3.6 Conversion vs. Time Curve Determined from SAXS Fitting

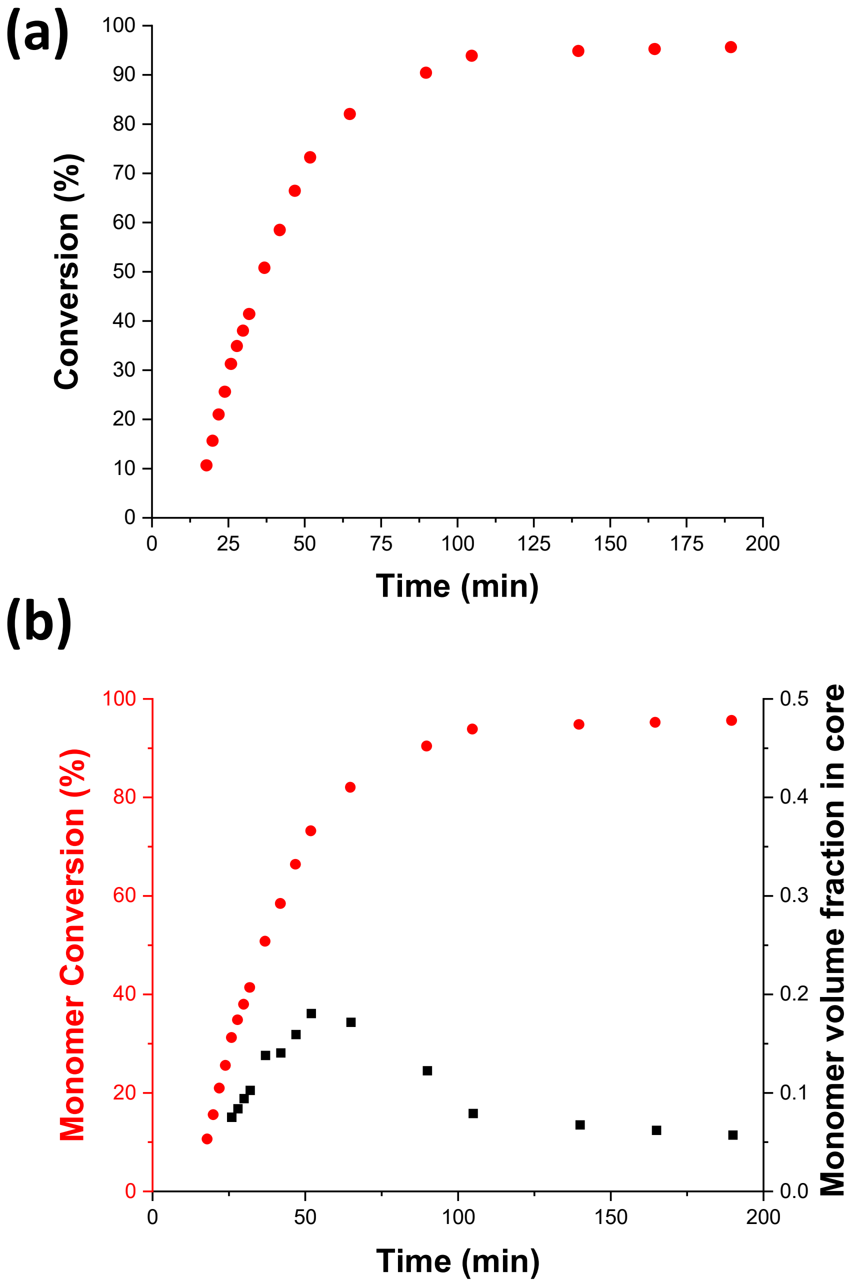

Figure S7. (a) TFEMA conversion vs. time curve obtained from data fits to the SAXS patterns when using the three-population scattering model combined with the population balance constraint. (b) TFEMA conversion vs. time curves (red circles) and volume fraction of monomer within the growing nanocomposite particle cores (black squares) obtained from SAXS data fits using the population balance model.

3.7 Comparing Particle Size determined by DLS and *in situ* SAXS

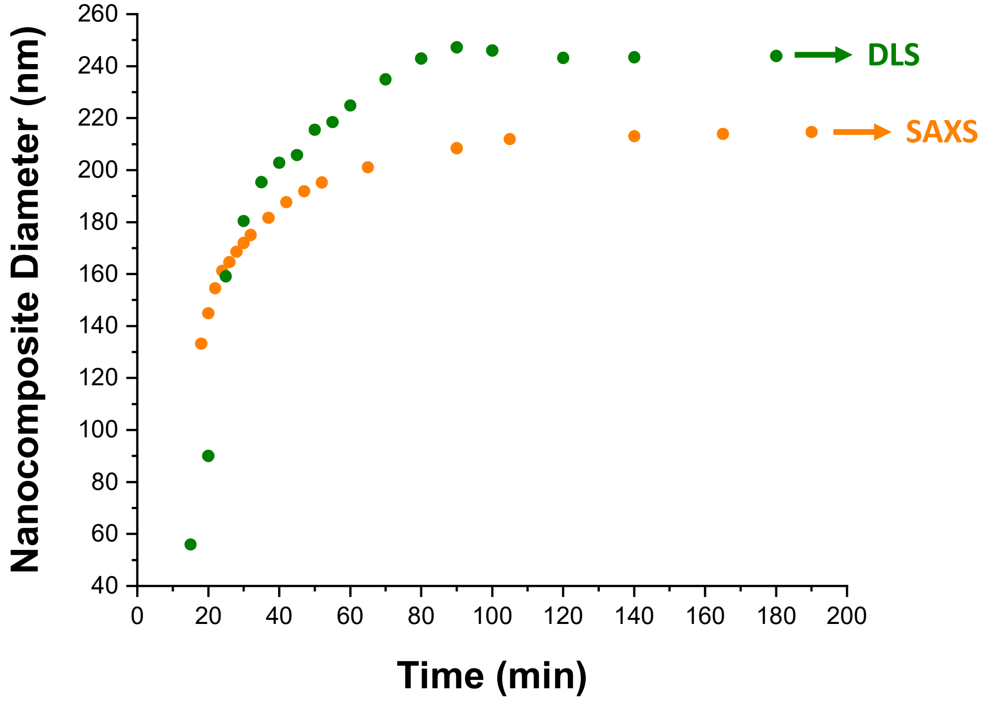

Figure S8. Evolution in the volume-average diameter over time determined by *in situ* SAXS and DLS respectively during the corresponding laboratory-scale synthesis of PTFEMA/silica nanocomposite particles via the aqueous emulsion polymerization of TFEMA at 60 °C in the presence of the glycerol-functionalized silica nanoparticles using a cationic azo initiator and targeting 10% w/w solids.

3.8 Silica shell SLD

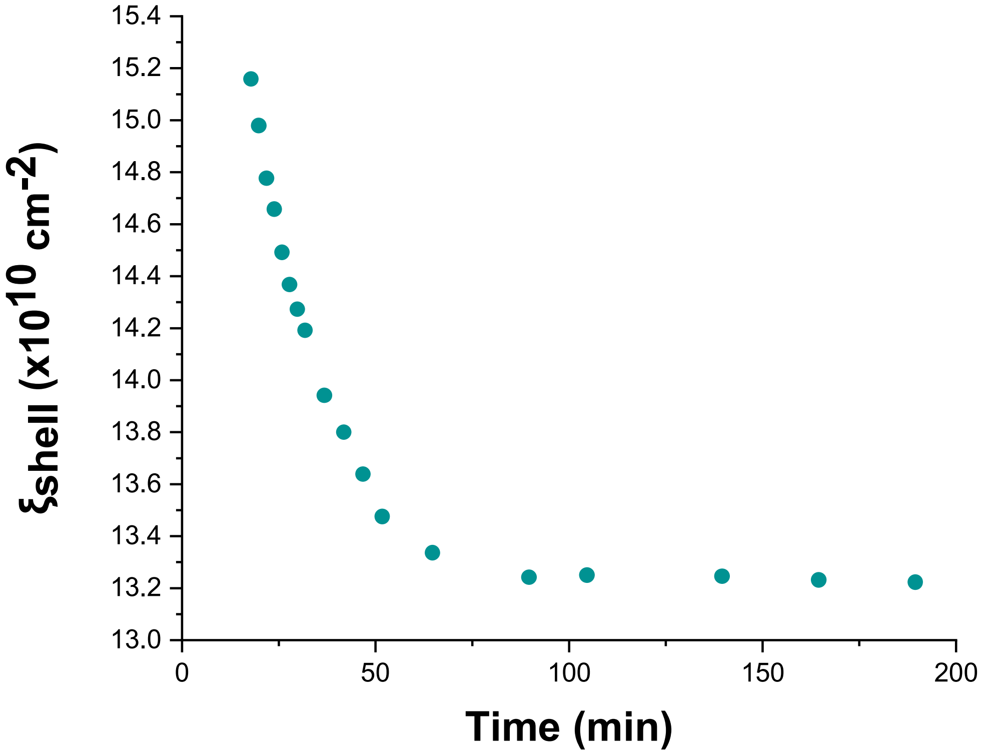

Figure S9. Change in the apparent scattering length density (SLD) for the silica shell over time determined by SAXS analysis during the aqueous emulsion polymerization of TFEMA in the presence of glycerol-functionalized silica nanoparticles at 60 °C using a cationic azo initiator when targeting 10% w/w solids.

3.9 Silica shell packing density, ϕ_{silica}

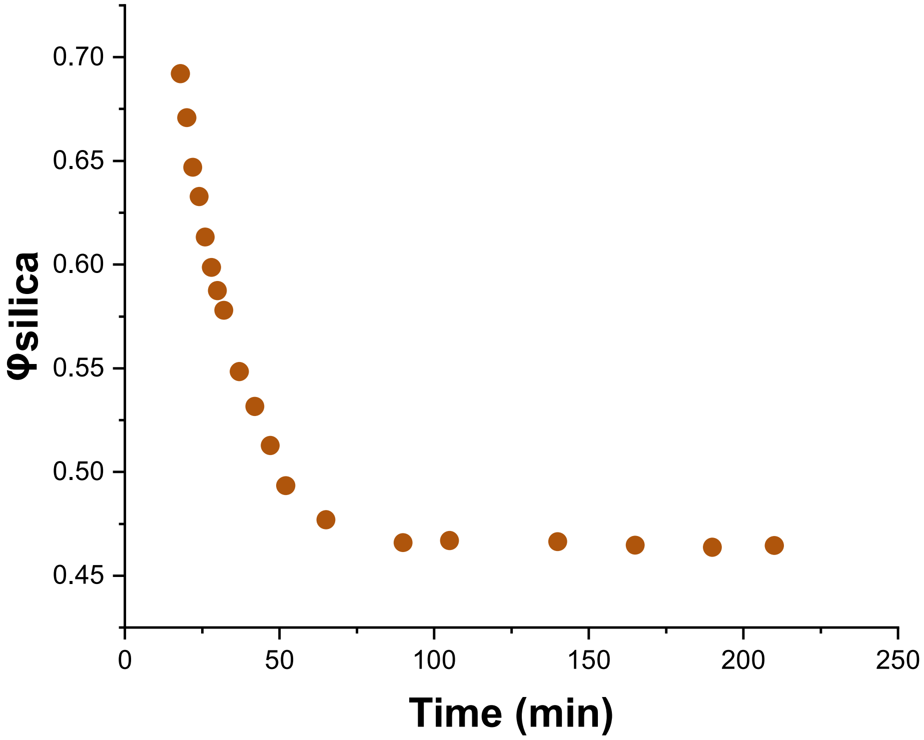

Figure S10. Change in the apparent packing density (ϕ_{silica}) of silica nanoparticles within the silica shell determined during the TFEMA polymerization using equation (S5).

3.10 Optical Microscopy Images

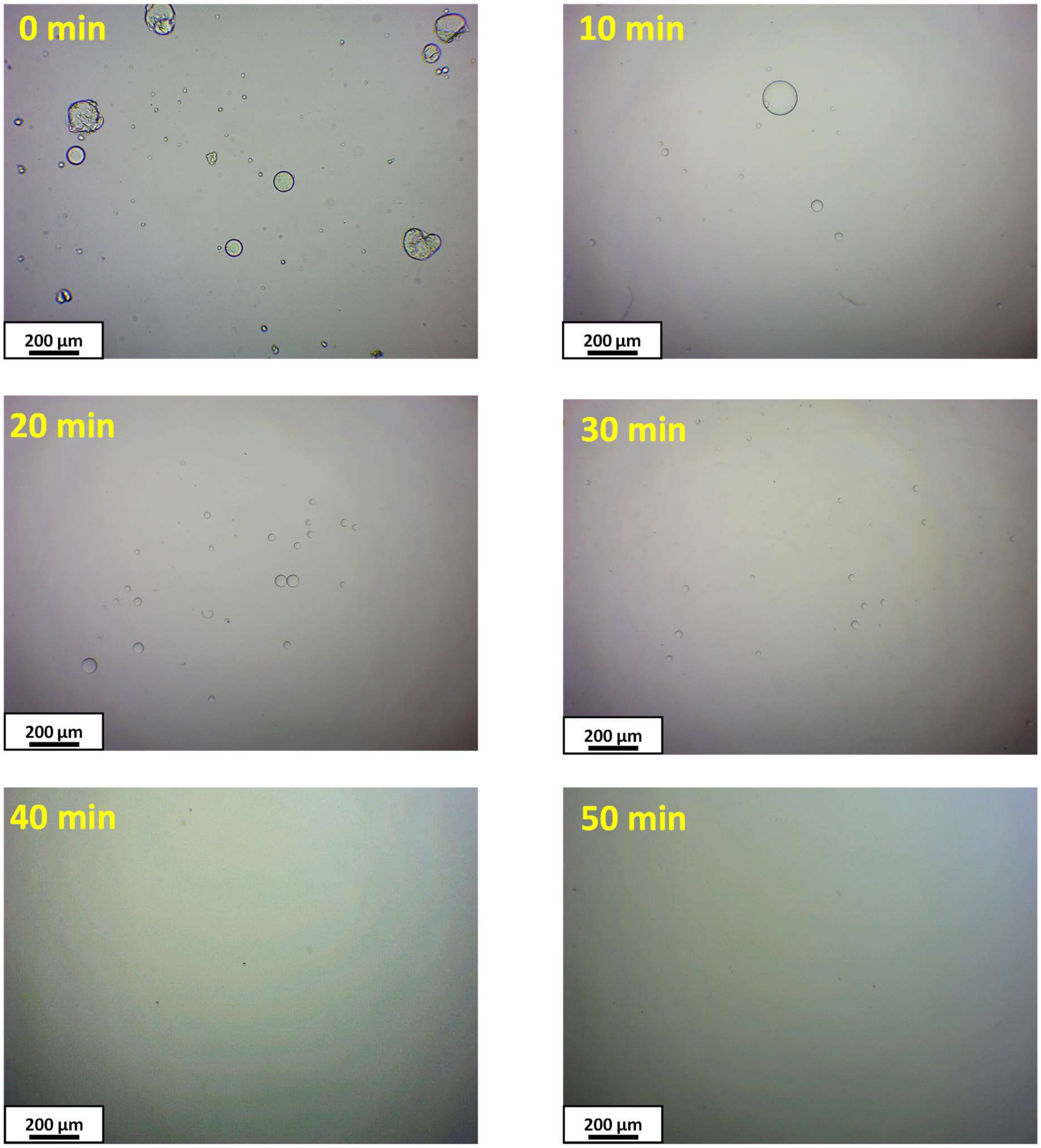

Figure S11. Optical microscopy images recorded at various time points during the laboratory-based synthesis of PTFEMA nanocomposite particles via aqueous emulsion polymerization of TFEMA in the presence of glycerol-functionalized silica nanoparticles at 60 °C when targeting 10% w/w solids.

3.11 Scattering Patterns during Polymerization

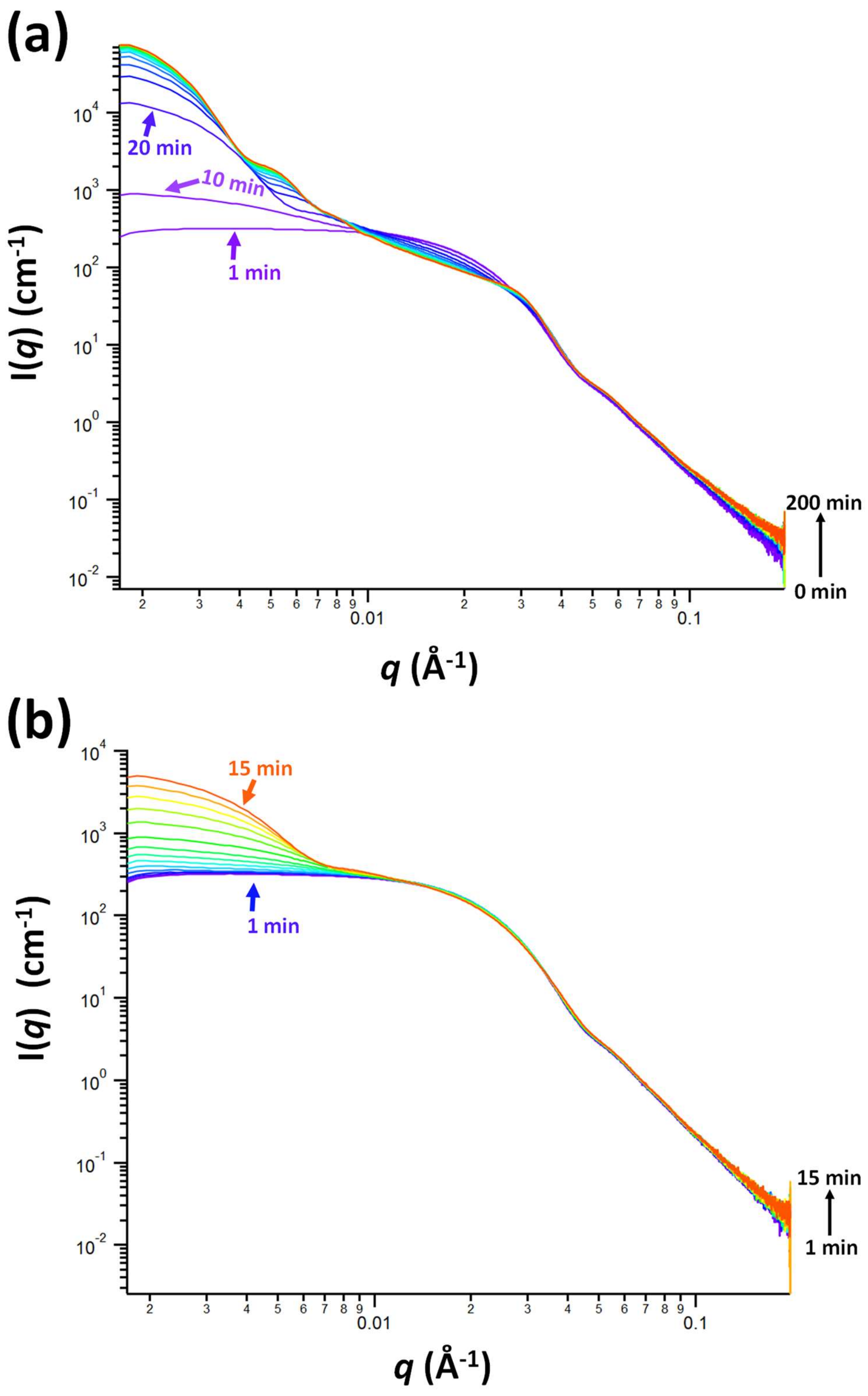

Figure S12. (a) Selected scattering patterns recorded during the whole polymerization (i.e. over 200 min), and (b) Selected scattering patterns recorded during the first 15 min.

3.12 Scattering Patterns Prior to Nucleation

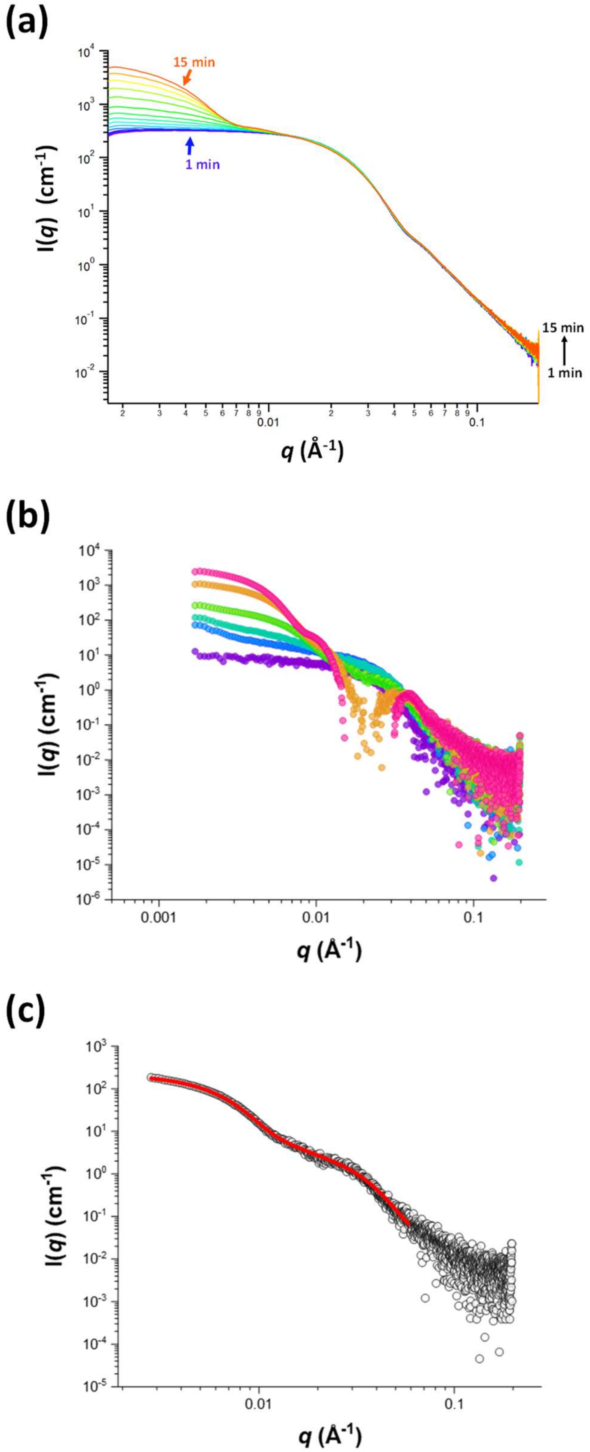

Figure S13. (a) Selected scattering patterns recorded during the first 15 min of polymerization with water subtracted as the background, (b) Scattering patterns recorded during the first 15 min of polymerization with frame 1 (i.e. silica nanoparticles) subtracted as the background, and (c) Scattering pattern recorded after 8 min - also shown in part (b) - fitted using a simple sphere model (see red solid line). This fit indicates a volume-average diameter of 63 nm.

4. Scattering Model for SAXS analysis

As outlined in the main text, a three-population scattering model is used to fit the *in situ* SAXS patterns recorded for the PTFEMA/silica nanocomposite particles, which acquire a well-defined core-shell morphology after a certain time point.

The scattered intensity I(q) is measured as a function of the scattering vector length $q = (4\pi/\lambda)\sin\theta$, where 2θ is the scattering angle. After subtraction of the solvent background, the scattered intensity (or differential scattering cross-section per unit sample volume) for a colloidal dispersion can be approximately expressed as:

$$\mathrm{I}(q) = \sum_{\mathrm{i}=1}^{n} S_{\mathrm{i}}(q) N_{\mathrm{i}} \int_0^{\infty} \left[F_{\mathrm{i}}^{\mathrm{pop}}(q,r)\right]^2 \Psi_{\mathrm{i}}(r)\ \mathrm{d}r \qquad \text{(S2)}$$

where *n* is the number of types of particles present in the dispersion, $S_{\mathrm{i}}(q)$ is the structure factor arising from interparticle interactions, N_{i} is the number density of scattering particles of the $i\mathrm{th}$ population per unit sample volume, $F_{\mathrm{i}}^{\mathrm{pop}}(q,r)$ is the scattering amplitude that describes the particle morphology, and $\Psi_{\mathrm{i}}(r)$ is the size distribution function of scattering particles corresponding to the ith population.

The silica shell within the nanocomposite particles is *particulate* in nature and excess (non-adsorbed) silica nanoparticles are present in the aqueous continuous phase throughout polymerization. Thus the scattering intensity [eq (S2)] is best analyzed using a three-population model (*n* = 3). The first population (*i* = 1) describes the core/shell morphology of the PTFEMA/silica nanocomposite particles. However, this merely assumes a homogenous distribution of electron density within the shell layer. Thus, a second population (*i* = 2) describing the *particulate* nature of this shell is also required.[1] The third population (*i* = 3) is used to account for the gradual reduction in concentration of the non-adsorbed silica

nanoparticles that remain within the aqueous continuous phase as the TFEMA polymerization progresses.

The following functions and parameters were used for the three-population scattering model.

Population 1 *– Core/shell nanocomposite particles*

The scattering amplitude of the core/shell nanocomposite particles can be described as follows:

$$F_1^{\text{pop}}(q,r) = V(r+S_\text{t})(\xi_\text{shell} - \xi_\text{med})\, f[q(r+S_\text{t})] + V(r)(\xi_\text{core} - \xi_\text{shell}) f(qr) \quad \text{(S3)}$$

where $V(x) = {}^4\!/_3\,\pi x^3$, $f(z) = 3(\sin z + z\cos z)/z^3$ is the normalized scattering amplitude of a homogeneous sphere and S_t is the shell thickness. ξ_med, and ξ_core and ξ_shell are the scattering length densities of the medium, the PTFEMA core and the silica shell, respectively. Since the shell is composed of water molecules and silica nanoparticles, the (averaged) scattering length density of the shell can be expressed as:

$$\xi_\text{shell} = \phi_\text{silica} \cdot \xi_\text{silica} + (1 - \phi_\text{silica}) \cdot \xi_{\text{H}_2\text{O}} \quad \text{(S4)}$$

where ξ_silica is the scattering length density of the silica nanoparticles, $\xi_{\text{H}_2\text{O}}$ is the scattering length density of water, and ϕ_silica is the packing efficiency of the silica nanoparticles within the shell, which is defined as:

$$\phi_\text{silica} = \frac{A_\text{e} \cdot v_\text{silica}}{A_\text{e} \cdot v_\text{silica} + v_\text{sol-shell}} \quad \text{(S5)}$$

where v_silica is the relative volume fraction of silica in the system, i.e. both free silica nanoparticles [(1-A_e)v_silica] and that which forms the silica shell ($A_\text{e}v_\text{silica}$), A_e is the fraction of

adsorbed silica nanoparticles (or the silica aggregation efficiency), and $v_{\text{sol-shell}}$ is the relative volume fraction of water within the silica shell.

Initially, the TFEMA monomer mostly resides in relatively large (*ca*. 50 µm diameter) monomer droplets, with only a small fraction dissolved within the aqueous continuous phase. However, after nucleation, monomer diffuses into the growing particles to solvate the PTFEMA chains and enable the polymerization to continue. Therefore, the scattering length density of the latex core (equation S6) is defined by the scattering length densities of the monomer and polymer and is described as:

$$\xi_{\text{core}} = xmon \cdot \xi_{\text{mon}} + (1 - xmon) \cdot \xi_{\text{pol}} \quad \text{(S6)}$$

where $xmon$ is the volume fraction of TFEMA in the polymer cores. It was also assumed that the water content within such hydrophobic cores is negligible. Because the aqueous solubility of TFEMA monomer is relatively low, it makes no contribution to the scattering length density of the aqueous continuous phase (ξ_{med}). Thus

$$\xi_{\text{med}} = \xi_{H_2O} \quad \text{(S7)}$$

To a good approximation, the polydispersity of the core/shell nanocomposite particles can be described by the polydispersity of the PTFEMA latex core radius. This is expressed in terms of a Gaussian distribution:

$$\Psi_1(r) = \frac{1}{\sqrt{2\pi\sigma_{R_\text{c}}{}^2}} e^{-(r-R_\text{c})^2/2\sigma_{R_\text{c}}{}^2} \quad \text{(S8)}$$

where R_c is the mean latex core radius and σ_{R_c} is its corresponding standard deviation.

Clearly, the mass of silica nanoparticles and the total mass of TFEMA/PTFEMA both remain constant during polymerization. Thus several constraints can be incorporated in the model.

The ratio between the total volume of the shell, $\int_0^{\infty}[V(r+S_\mathrm{t})-V(r)]\Psi_1(r)\,\mathrm{d}r$, and the total volume of the core, $\int_0^{\infty}V(r)\Psi_1(r)\,\mathrm{d}r$, can be expressed by the ratio of volume fractions of the components comprising the particle shell and core:

$$\frac{\int_0^{\infty}[V(r+S_\mathrm{t})-V(r)]\Psi_1(r)\,\mathrm{d}r}{\int_0^{\infty}V(r)\Psi_1(r)\,\mathrm{d}r}=\frac{A_\mathrm{e}\cdot v_\mathrm{silica}+v_\mathrm{sol-shel}}{\frac{\rho_\mathrm{mon}}{\rho_\mathrm{pol}}\cdot conv\cdot v_\mathrm{mon}}\cdot(1-xmon) \tag{S9}$$

where v_mon is the initial volume fraction of the monomer, *conv* is the monomer conversion, and ρ_mon and ρ_pol are the densities of TFEMA monomer and PTFEMA at the synthesis temperature, respectively. The left-hand term in equation S9 can be approximated by the ratio of the average shell volume, $V_\text{sh-avg}$, and the average core volume, $V_\text{co-avg}$, and hence rewritten as:

$$\frac{V_\mathrm{sh-av}}{V_\mathrm{co-a}}\approx\frac{A_\mathrm{e}\cdot v_\mathrm{silica}+v_\mathrm{sol-shel}}{\frac{\rho_\mathrm{mo}}{\rho_\mathrm{pol}}\cdot conv\cdot v_\mathrm{mon}}\cdot(1-xmon) \tag{S10}$$

where $\frac{V_\mathrm{sh-a}}{V_\mathrm{co-avg}}=\frac{(S_\mathrm{t}+R_\mathrm{c})^3}{R_\mathrm{c}^3}-1.$

Thus the shell thickness can be expressed using equation S10 via the fraction of aggregated silica nanoparticles:

$$S_\mathrm{t}\approx R_\mathrm{c}\left\{\left[\frac{A_\mathrm{e}\cdot v_\mathrm{silica}\cdot(1-xmon)}{\phi_\mathrm{silica}\cdot\frac{\rho_\mathrm{mon}}{\rho_\mathrm{pol}}\cdot conv\cdot v_\mathrm{mon}}+1\right]^{\frac{1}{3}}-1\right\} \tag{S11}$$

The number density of the core/shell nanocomposite particles, N_1, for equation S2 can be expressed as:

$$N_1 = \frac{\frac{\rho_{mon}}{\rho_{pol}} \cdot \frac{conv \cdot v_{\text{mon}}}{1-xmo}}{\int_0^\infty V(r)\Psi_1(r)\,\mathrm{d}r} \quad \text{(S12)}$$

Owing to the relatively high targeted solids concentration (10% w/w), it is necessary to account for interactions between the core/shell particles. Thus a hard-sphere structure factor (solved using the Percus-Yevick closure relation), $S_1(q, D_{\text{csPY}}, \Phi_{\text{csPY}})$, was incorporated in the scattering equation (equation S2), where D_{csPY} is the distance between the centres of the core/shell nanocomposite particles and Φ_{csPY} is the effective volume fraction of these particles.

Population 2 *– Silica particles located within the nanocomposite shell*

The form factor for the adsorbed silica nanoparticles located within the shell, F_2^{pop}, can be described by the following equation:

$$F_2^{\text{pop}}(q,r) = V(r)(\xi_{\text{silica}} - \xi_{\text{sol}})f(qr) \quad \text{(S13)}$$

where $V(r)$ corresponds to the silica nanoparticle volume. The Gaussian size distribution of the silica nanoparticles within the shell in equation S2 is described by the following expression:

$$\Psi_2(r) = \frac{1}{\sqrt{2\pi}\sigma_{R_{\text{silica}}}} e^{-(r-R_{\text{silica}})^2/2\sigma_{R_{\text{silica}}}{}^2} \quad \text{(S14)}$$

where R_{silica} is the mean silica nanoparticle radius and $\sigma_{R_{\text{silica}}}$ is the standard deviation. Both parameters are determined from the first frame of the time-resolved SAXS patterns recorded during synthesis (Figure S4).

The number density of the silica nanoparticles, N_2, for equation S2 can be expressed as:

$$N_2 = \frac{A_{\text{e}} \cdot v_{\text{silica}}}{\int_0^\infty V(r)\Psi_2(r)\,\mathrm{d}r} \quad \text{(S15)}$$

A structure factor, $S_2(q, D_{sPY}, \Phi_{sPY})$, in equation S2 accounts for the relatively dense packing of the silica nanoparticles within the shell. This S_2 term corresponds to a hard-sphere structure factor that is solved using the Percus-Yevick closure relation, where D_{sPY} is the distance between the centres of the silica nanoparticles and Φ_{sPY} is the effective volume fraction of packed silica nanoparticles.

Population 3 – *Non-adsorbed excess silica nanoparticles located within the aqueous phase*

The scattering amplitude of the non-adsorbed (free) silica nanoparticles located throughout the aqueous phase can be described as follows:

$$F_3^{\text{pop}}(q,r) = V(r)(\xi_{\text{silica}} - \xi_{\text{sol}})f(qr) \tag{S16}$$

where $V(r)$ corresponds to the silica nanoparticle volume. The Gaussian size distribution of the silica nanoparticles is described in equation S2 by the same parameters used for population 2 (see equation S14):

$$\Psi_3(r) = \frac{1}{\sqrt{2\pi\sigma_{R_{\text{silica}}}{}^2}} e^{-(r-R_{\text{silica}})^2/2\sigma_{R_{\text{silica}}}{}^2} \tag{S17}$$

The number density of silica nanoparticles located throughout the continuous phase is described in equation S2 as:

$$N_3 = \frac{(1-A_{\text{e}})\cdot v_{\text{silica}}}{\int_0^{\infty} V(r)\Psi_3(r)\,\text{d}r} \tag{S18}$$

Because the concentration of excess silica nanoparticles is relatively low, it is assumed that there are no interparticle interactions, hence $S_3(q) = 1$ in equation S2.

Number of silica nanoparticles located within the shell of the core-shell nanocomposite particles

The mean number of silica nanoparticles (P_s) can be calculated from the number density of adsorbed silica nanoparticles (equation S15) and the number density of core/shell nanocomposite particles (equation S12):

$$N_2 = P_s \cdot N_1 \quad \text{(S19)}$$

If the ratio of the total PTFEMA core volume to the total volume of the aggregated silica nanoparticles can be approximated by the cube of the ratio of the mean PTFEMA core radius to the mean silica nanoparticle radius, then the mean number of silica nanoparticles within the shell can be calculated using:

$$P_s = \phi_{\text{silica}} \frac{[(S_t + R_c)^3 - R_c{}^3]}{R_{\text{silica}}{}^3} \quad \text{(S20)}$$

SAXS analysis

During SAXS analysis, it is assumed that the silica volume fraction (v_{silica}), the silica nanoparticle radius and polydispersity (R_{silica}, σ_{silica}), the total mass of TFEMA and PTFEMA, and the mass densities and scattering length densities of the various components [water (ρ_{H2O} and ξ_{H_2O}), monomer (ρ_{mon} and ξ_{mon}), polymer (ρ_{pol} and ξ_{pol}) and silica (ρ_{silica} and ξ_{silica})] all remain constant during the polymerization. For the first SAXS pattern recorded prior to any polymerization, the scattering is dominated by the silica nanoparticles because the TFEMA monomer droplets are so large they lie outside of the experimentally accessible q range (Figure S4). Thus this initial scattering pattern was used to determine R_{silica}, σ_{silica} and v_{silica}. Here only population 3 was included in the fitting process and A_e = 0 (i.e. all silica

nanoparticles are located within the aqueous continuous phase). The last frame recorded during the time-resolved SAXS experiment corresponds to the end of the TFEMA polymerization: this was used to define the initial relative volume fraction of TFEMA monomer (v_{mon}) assuming a final conversion (*conv*) of 96%, as determined by *postmortem* ^{1}H NMR studies. All three populations in equation S2 were included during the fitting process, see Figure S5. Thus, all the intermediate SAXS patterns were analyzed using ten independent fitting parameters (R_c, σ_c, D_{csPY}, Φ_{csPY}, $xmon$, $conv$, A_e, ϕ_{silica}, D_{sPY}, Φ_{sPY}) and the twelve fixed parameters summarized in Table S1. Our approach was to analyze the penultimate frame and continue backwards in terms of the reaction time until a SAXS pattern was identified for which the three-population model was no longer appropriate. This three-population model is not expected to be valid during the early stages of polymerization because well-defined core/shell nanocomposite particles are not formed until well after the nucleation event. The other variables associated with the structural model are dependent on the following six parameters, which are fitted:

$$\xi_{\text{shell}}\left(\phi_{\text{silica}}, \xi_{\text{silica}}, \xi_{H_2O}\right) \quad \text{(from equation S4)}$$

$$v_{\text{sol-shell}}\left(A_e, \phi_{\text{silica}}, v_{\text{silica}}\right) \quad \text{(from equation S5)}$$

$$\xi_{\text{core}}\left(xmon, \xi_{\text{mon}}, \xi_{\text{pol}}\right) \quad \text{(from equation S6)}$$

$$\xi_{\text{med}}\left(\xi_{\text{H}_2\text{O}}\right) \quad \text{(from equation S7)}$$

$$S_{\text{t}}\left(R_{\text{c}}, conv, v_{\text{mon}}, v_{\text{silica}}, A_{\text{e}}, \phi_{\text{silica}}, xmon, \rho_{\text{mon}}, \rho_{\text{pol}}\right) \quad \text{(from equation S11)}$$

$$P_{\text{s}}\left(R_{\text{c}}, R_{\text{silica}}, conv, v_{\text{mon}}, v_{\text{silica}}, A_{\text{e}}, xmon, \rho_{\text{mon}}, \rho_{\text{pol}}\right) \quad \text{(from equation S20)}$$

Table S1. Summary of the twelve variables that are assumed to remain constant during the time-resolved SAXS experiment described in this study.

Constant variable	Symbol	Value used for the analysis
Mass density of each component	$\rho_{H2O}{}^{a}$	0.983 g·cm^{-3}
	$\rho_{mon}{}^{a}$	1.113 g·cm^{-3}
	$\rho_{pol}{}^{b}$	1.456 g·cm^{-3}
	$\rho_{silica}{}^{c}$	2.092 g·cm^{-3}
Relative silica concentration	$v_{silica}{}^{d}$	1.2% v/v
Silica particle radius and polydispersity	$R_{silica}{}^{d}$	9.75 nm
	$\sigma_{Rsilica}{}^{d}$	2.14 nm
SLD values for each component	ξ_{H2O}	9.271×10^{10} cm^{-2}
	ξ_{mon}	9.669×10^{10} cm^{-2}
	ξ_{pol}	1.265×10^{11} cm^{-2}
	ξ_{silica}	1.775×10^{11} cm^{-2}
Relative monomer concentration	$v_{mon}{}^{e}$	2.96% v/v

[a]Mass densities determined using an Anton Paar DMA 5000M densitometer at the polymerization temperature (60 °C).

[b]Density was measured by helium pycnometry (Micrometrics AccuPyc II 1340 instrument) at 20 °C and corrected for the polymerization temperature using the known thermal expansion coefficient for poly(methyl methacrylate).[2]

[c]Density was measured by helium pycnometry at 20 °C. Given that the relatively low linear thermal expansion coefficient of fused silica is 5.5×10^{-7} K^{-1}, this value was assumed to be valid at the polymerization temperature.[3]

[d]These parameters were determined by fitting the SAXS model to the first frame recorded during the time-resolved SAXS experiment (Figure S4).

[e]This parameter was determined by fitting the SAXS model to the final frame recorded during the time-resolved SAXS experiment (Figure S5).

Table S2. Summary of the sixteen variables used for data fits to the time-resolved SAXS patterns recorded in this study.

Fitted variable	Symbol
PTFEMA core radius	R_c
PTFEMA core radius standard deviation	σ_c
Distance between the centers of the silica nanoparticles	D_{csPY}
Volume fraction of core/shell nanoparticles	Φ_{csPY}
Volume fraction of TFEMA monomer within the latex core	$xmon$
TFEMA monomer conversion	*conv*
Aggregation efficiency of silica nanoparticles	A_e
Packing density of silica nanoparticles within the silica shell	ϕ_{silica}
Distance between the centers of the silica nanoparticles	D_{sPY}
Effective volume fraction of packed silica nanoparticles	Φ_{sPY}
Scattering length density of silica shell	ξ_{shell}
Volume of silica shell	$v_{sol\text{-}shell}$
Scattering length density of PTFEMA core	ξ_{core}
Scattering length density of medium	ξ_{med}
Silica shell thickness	S_t
Average number of silica nanoparticles within the shell	P_s

References

1 J. A. Balmer, O. O. Mykhaylyk, S. P. Armes, J. P. A. Fairclough, A. J. Ryan, J. Gummel, M. W. Murray, K. A. Murray and N. S. J. Williams, *J. Am. Chem. Soc.*, 2011, **133**, 826–837.

2 https://materials.springer.com/lb/docs/sm_nlb_978-0-387-69002-5_7.

3 B. J. Skinner, "SECTION 6: THERMAL EXPANSION", Handbook of Physical Constants, Sydney P. Clark, Jr., 1966.
